# Modulation of the Maladaptive Stress Response to Manage Diseases of Protein Folding

**DOI:** 10.1371/journal.pbio.1001998

**Published:** 2014-11-18

**Authors:** Daniela Martino Roth, Darren M. Hutt, Jiansong Tong, Marion Bouchecareilh, Ning Wang, Theo Seeley, Johanna F. Dekkers, Jeffrey M. Beekman, Dan Garza, Lawrence Drew, Eliezer Masliah, Richard I. Morimoto, William E. Balch

**Affiliations:** 1 Department of Cell Biology, The Scripps Research Institute, La Jolla, California, United States of America; 2 Department of Molecular Biosciences, Rice Institute for Biomedical Research, Northwestern University, Evanston, Illinois, United States of America; 3 Department of Pediatric Pulmonology, Wilhelmina Children's Hospital, University Medical Centre, Utrecht, The Netherlands; 4 Laboratory of Translational Immunology, Wilhelmina Children's Hospital, University Medical Centre, Utrecht, The Netherlands; 5 Proteostasis Therapeutics Inc., Cambridge, Massachusetts, United States of America; 6 Department of Neurosciences, University of California, San Diego, La Jolla, California, United States of America; 7 The Skaggs Institute for Chemical Biology, The Scripps Research Institute, La Jolla, California, United States of America; 8 Department of Chemical Physiology, The Scripps Research Institute, La Jolla, California, United States of America; 9 The Institute for Childhood and Neglected Diseases, The Scripps Research Institute, La Jolla, California, United States of America; Max Planck Institute for Biochemistry, United States of America

## Abstract

Diseases of protein folding arise because of the inability of an altered peptide sequence to properly engage protein homeostasis components that direct protein folding and function. To identify global principles of misfolding disease pathology we examined the impact of the local folding environment in alpha-1-antitrypsin deficiency (AATD), Niemann-Pick type C1 disease (NPC1), Alzheimer's disease (AD), and cystic fibrosis (CF). Using distinct models, including patient-derived cell lines and primary epithelium, mouse brain tissue, and *Caenorhabditis elegans*, we found that chronic expression of misfolded proteins not only triggers the sustained activation of the heat shock response (HSR) pathway, but that this sustained activation is maladaptive. In diseased cells, maladaptation alters protein structure–function relationships, impacts protein folding in the cytosol, and further exacerbates the disease state. We show that down-regulation of this maladaptive stress response (MSR), through silencing of HSF1, the master regulator of the HSR, restores cellular protein folding and improves the disease phenotype. We propose that restoration of a more physiological proteostatic environment will strongly impact the management and progression of loss-of-function and gain-of-toxic-function phenotypes common in human disease.

## Abstract 2

This study shows how chronic stress and heat shock response exacerbate the phenotype in protein misfolding diseases by triggering a Maladaptive Stress Response; this pathway represents a promising therapeutic target for multiple genetic disorders.

## Author Summary

The function of all proteins is dependent on achieving the correct folded state, a process referred to as protein homeostasis or proteostasis. Cellular proteostasis is maintained by diverse signaling pathways, including the heat shock response (HSR), which protects proteins in the face of acute stress. However, genetic disorders are a challenge to cells, since the mutated protein will often fail to fold properly and function correctly. We have discovered that the chronic expression of such disease-causing proteins can trigger the sustained activation of the HSR in a failed attempt to correct the associated misfolding defect. Such chronic HSR activation presents an unanticipated challenge to the cell by initiating a sustained state of stress management, which leads to a general protein-folding deficiency. This in turn further exacerbates the disease phenotype—a condition we have termed maladaptive. We show that down-regulation of this maladaptive stress response (MSR) restores cellular protein folding and improves the disease condition in loss-of-function disorders such as cystic fibrosis, Niemann-Pick disease and alpha-1-antitrypsin deficiency, as well as gain-of-toxic-function diseases such as Alzheimer's disease. MSR management therefore potentially represents an important therapeutic first step in regulating the progression of human disease associated with chronic protein misfolding.

## Introduction

The transition from protein folding to misfolding, in both normal physiology and disease, is dynamically managed by multiple proteostatic pathways [1],[2]. The heat shock response (HSR) is a central signaling pathway managing the malleable composition of the proteostasis network (PN) of folding and degradation machineries. The cellular PN environment contributes to what we refer to as the quinary (Q) state of the protein fold [3]–[6], which emphasizes that the structure of a protein is tightly integrated with a dynamic proteostatic system to direct structure–function relationships in health and address challenges in response to disease [1],[5],[71]–[11]. Q-state managers of each protein fold draw from the proteostasis pool of molecular chaperones (Hsp40s, Hsc70s, Hsp70s, and Hsp90), small heat shock proteins, and ubiquitin-based degradation components [3],[5],[12]–[14]. These managers are responsive to multiple signaling pathways including the unfolded protein response (UPR) [15], controlling compartmentalized folding, and the heat-shock response (HSR), controlling cytoplasmic/nuclear folding [8]. The importance of an integrated Q-state is exemplified in the function of coupled protein synthesis and folding machineries [16], linked cargo-specific folding and trafficking machineries [1],[5],[7]–[9],[17], and the activity of cytoplasmic Q-bodies that actively monitor the health of each protein in the cytoplasm [6],[18]. Together, these proteostasis machineries operate as integrated sensors of individual protein structure–function relationships that now need to be understood [3],[6],[19]–[24].

The HSR is controlled by the heat shock transcription factor 1 (HSF1), with the chaperone Hsp90 regulating its activation [8],[25]. Transient stimulation of the HSR pathway, based on the heat shock paradigm [26], is generally beneficial in that it alters the composition of proteostasis components in the cytosol to provide immediate, but temporary, protection to misfolded proteins in the face of divergent stress insults [8],[9],[27]. Consistent with this view, transcriptional profiling, in response to acute heat shock, revealed that approximately 500 genes are up-regulated, whereas more than 1,000 genes are repressed [28]–[30], conditions that, if sustained, could negatively impact cell viability. Our understanding of these complex gene expression changes and their impact on protein structure–function relationships in response to chronic folding insults remains to be elucidated.

Diseases of protein folding arise due to the inability of an altered peptide sequence to properly engage the prevailing local proteostasis components. Gain-of-toxic-function diseases such as Alzheimer's (AD) [31] and inherited loss-of-function diseases such as alpha-1-antitrypsin deficiency (AATD) [32],[33], Niemann-Pick type C1 disease (NPC1), and cystic fibrosis (CF) [34] present a unique challenge to cells because of the chronic nature of the insult [34],[35]. A current paradigm in disease biology is that stress pathways are not sufficiently activated to provide the necessary protection. Therefore, activation of these pathways, such as the HSR, should improve folding and/or clearance of disease-related proteins. Indeed, HSF1 activation has been shown to provide partial correction for some misfolding diseases [36], however, the in vivo benefits for the chronic activation of HSF1 have not been investigated. Recently, HSR activation has been shown to exacerbate the aggregation of mutant huntingtin protein (htt-Q91) in a cellular model of Huntington's disease (HD) [37]. Moreover, sustained HSR activation promotes proliferation of cancer cells [28],[38], a pathologic disease state leading to reduced human lifespan. In cancer cells, HSF1 drives a distinct transcriptional program from the classical HSR, implying a more complex function than previously anticipated [39]. We have recently suggested that HSF1 activators that partially promote correction of CF do so by activation of unknown cellular pathways [40], which we now need to understand in the context of the prevailing proteostasis biology to provide new insights into the evolution of chronic disease management by the cell [1],[5].

Herein we have studied four misfolding disorders to address central principles in managing chronic protein folding stress in human disease: (1) the deletion of phenylalanine 508 (F508del) variant of the cystic fibrosis transmembrane conductance regulator (CFTR) (F508del-CFTR), a multi-membrane–spanning protein with large cytoplasmic domains, which fails to traffic to the plasma membrane and is responsible for 90% of CF cases [10],[34]; (2) the Z-variant of alpha-1-antitrypsin (Z-AAT), which accumulates as a misfolded polymer in the early secretory endoplasmic reticulum (ER) compartment, leading to liver disease and chronic obstructive pulmonary disease (COPD)/emphysema because of its failure to be secreted and delivered to the lung [32],[33]; (3) the I1061T variant of NPC1, key component in lipid and cholesterol homeostasis in the late endosome/lysosome (LE/L) compartment, which fails to traffic from the ER to the LE/L in human disease, resulting in the lysosomal storage disease NPC1; and (4) AD, which arises from aberrant Alzheimer precursor protein (APP) processing and trafficking, resulting in accumulation of extracellular Aβ amyloid aggregates [31],[41].

Although our primary focus is on the correction of CF disease, we now show that the long-term expression of disease-causing misfolded proteins results in what we refer to as a maladaptive stress response (MSR), a state reflecting the sustained activation of the HSR pathway, which contributes to disease progression by undermining the normal folding capacity of cells. We provide evidence that silencing of HSF1 alleviates the MSR and improves the multiple disease phenotypes, suggesting a general principle that chronic alteration of the prevailing PN contributes to the progression of inherited diseases, a step that will now require active management to mitigate pathophysiology [1],[6].

## Results

### Chronic Proteotoxic Stress in CF Negatively Impacts Protein Folding

CF is caused by mutations in the multi-membrane–spanning protein CFTR, a chloride channel responsible for ionic and fluid homeostasis in the lung [34]. The F508del variant of CFTR is characterized by misfolding, ER accumulation, and removal by ER-associated degradation (ERAD) [34]. F508del-CFTR is retained in the ER in a Hsp70/90-containing chaperone trap, a step that wild-type (WT)-CFTR and temperature-corrected F508del (30°C) are able to navigate [42]. We therefore focused our attention on the HSR pathway that manages cytoplasmic chaperone biology.

**Figure 1:**
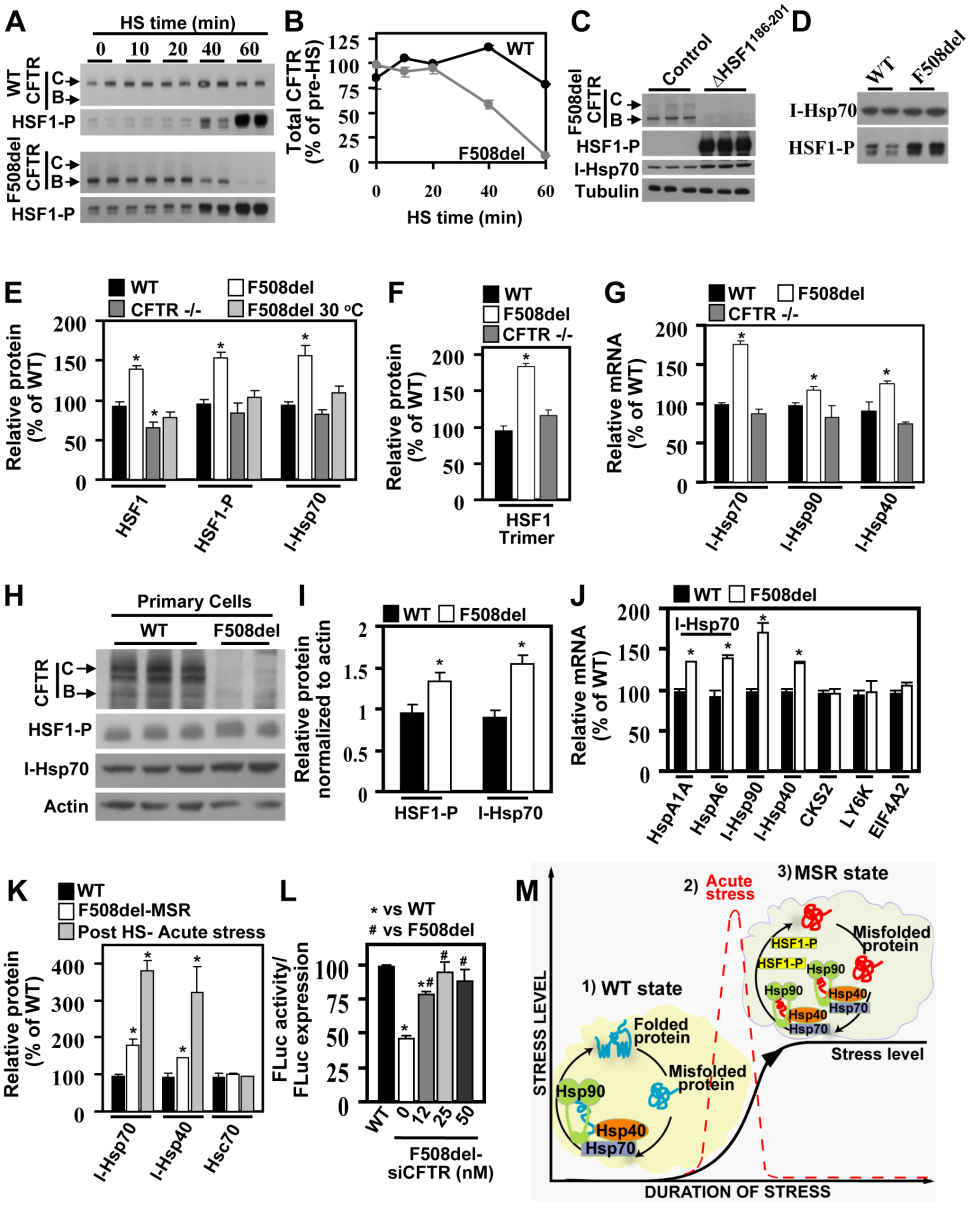
Expression of F508del induces chronic proteotoxic stress that affects cellular protein folding. (A) Immunoblots of WT-CFTR, F508del-CFTR, and HSF1-P during HS time course (42°C for a total of 60 min). (B) Quantification of total CFTR during HS, relative to pre-HS (T = 0) (*n* = 4). (C) Immunoblots of indicated proteins in CFBE41o- cellular lysates following the co-expression of F508del-CFTR with the constitutively active ΔHSF1^186–201^ or control empty plasmid. (D) Immunoblots of HSF1-P and I-Hsp70 in WT or F508del expressing cells (*n* = 4). (E) Quantification of the expression of HSR markers in WT-CFTR, F508del-CFTR at 37°C or 30°C, and CFTR null (CFTR−/−) expressing cells. (F) Quantification of HFS-1 trimer levels in WT-, F508del- and CFTR null–CFBE cells. (E,F) Results are shown as percentage of that seen in WT-expressing cells, as a mean ± standard error of the mean (SEM), n≥3. qRT-PCR of I-Hsp70 (HspA1A, or HspA6), I-Hsp90 (Hsp90α), and I-Hsp40 (DNAJB1) in (G,J) or of cancer-related HSF1 responsive genes (CKS2, LY6K, EIF4A2) shown in (J) from mRNA isolated from WT-, F508del- and CFTR null–CFBE cells (G) or from mRNA obtained from hBE primary cells obtained from homozygous patients for WT- or F508del-CFTR (J). qRT-PCR data was normalized to the housekeeping gene beta-glucuronidase (GUS). Results are shown as percentage of WT-expressing cells set to a 100 (mean ± standard deviation [SD] or SEM, n≥3, and * indicates *p*<0.05 relative to WT). Immunoblot (H) and quantification (I) of CFTR, HSF1-P, and I-Hsp70 from hBE primary cell lysates of WT or F508del patients. Data is shown as the relative protein expression normalized to actin (mean ± SD, n≥2). (K) Quantification of I-Hsp70 and I-Hsp40 protein level in F508del-expressing cells at 37°C (MSR) or following acute HS (shown as a percentage of the level seen in WT-expressing cells; mean ± SD, n≥2). (L) Firefly luciferase (FLuc) activity in WT- and F508del-CFTR expressing cells following siCFTR silencing. Results represent normalized specific activity of FLuc (luminescence/relative FLuc expression) for each condition. Data is shown as percentage of WT-CFTR expressing cells, mean ± SEM, n≥3, and *, # indicate *p*<0.05 relative to WT and F508del (0 nM siCFTR), respectively. (M) (1) Diagram showing the proteostatic environment of WT folding, where substrates are properly managed by the physiological Q-state (yellow cloud). (2) Representation of the transient stress level observed during acute stress responses (dotted red line). (3) The MSR state induced in misfolding diseases (abnormal Q-state, gray cloud) that results in a continuous elevated (subacute) stress affecting global folding and cellular function. All experimental data was repeated at least once. The underlying data used to make (B), (E–G), and (I–L) in this figure can be found in the supplementary file Data S1.

To assess the effect of HSR activation on the folding environment, we first heat shocked bronchial epithelial cells (CFBE41o-) expressing WT- or F508del-CFTR and monitored its impact on CFTR protein stability and trafficking. CFTR stability and trafficking is monitored by Western blot, in which the ER-localized (band-B) and post-ER glycoforms (band-C) exhibit a differential migration pattern. Whereas WT-CFTR remained mostly unaffected, more than 90% of F508del-CFTR was degraded after 60 min of heat shock (HS) (Figure 1A,B). HS activation was confirmed by increased HSF1 phosphorylation of Serine-326 (HSF1-P at S^326^). Since F508del-CFTR is sensitive to alterations in temperature, we determined whether the destabilization of F508del was caused by HSR activation and not simply elevated temperature. For this purpose, we overexpressed a constitutively active variant of HSF1 (ΔHSF1^186–201^) [43],[44] with F508del-CFTR in CFBE41o- cells. Overexpression of active ΔHSF1^186–201^, confirmed by elevated levels of HSF1-P and the stress-inducible Hsp70 (I-Hsp70), also led to destabilization of F508del-CFTR (Figure 1C). These data support the conclusion that activation of the HSR pathway results in destabilization of F508del-CFTR rather than correcting the stability and trafficking defect associated with this disease variant. We also observed that in the absence of HS, F508del-expressing cells already exhibited increased HSF1-P relative to that seen in WT-expressing cells (Figure 1A, 1D), revealing that the HSR pathway was already hyperactive in these cells. To confirm this observation, we compared additional markers of HSF1 activation, including HSF1 trimerization and expression of I-Hsp70. Cells expressing F508del-CFTR exhibited a significant increase in total, trimerized, and phosphorylated HSF1, as well as increased I-Hsp70 levels relative to WT-expressing cells (Figure 1D–1F). We also observed a significant increase in mRNA levels of the HSF1-responsive genes, HspA1A (I-Hsp70), Hsp90α (I-Hsp90), and DNAJB1 (I-Hsp40), relative to that seen in WT-expressing and in isogenic cells lacking CFTR (CFTR −/−) (Figure 1G). Additionally, silencing of F508del-CFTR led to a significant decrease in HSF1 and HSF1-P expression (Figure S1A, S1B), suggesting that the observed HSR activation is directly related to the expression of this misfolded CFTR variant. Temperature correction of F508del, which corrects its associated stability and trafficking defects, also led to a reduction in HSF1 and HSF1-P to levels seen in WT-expressing and CFTR−/− cells (Figure 1E). Altogether, our results suggest that the HSR activation observed in F508del-expressing cells at physiological temperature is a direct consequence of the expression of the misfolded F508del-CFTR.

To address whether the observed HSR activation was in response to the immortalized CFBE41o- cell line phenotype, we also examined these markers on patient-derived human bronchial epithelia (hBE) homozygous for WT- or F508del-CFTR. Consistent with the findings observed in cystic fibrosis bronchial epithelial (CFBE) cells, F508del-expressing hBE cells also showed elevated HSF1-P and I-Hsp70 protein levels, as well as increased I-Hsp70 (HspA1A & A6), I-Hsp90, and I-Hsp40 mRNA levels, relative to that seen in WT-expressing hBEs (Figure 1H–1J). No differences were observed in mRNA levels of non-classical HSF1-responsive genes, previously shown to be increased in cancer cells (CKS2, LY6K, and EIF4A2) (Figure 1J) [28], suggesting activation of the classical HSF1 pathway. In order to quantify the magnitude of this HSR activation, we compared the up-regulation of the I-Hsp40 and I-Hsp70 protein levels seen in F508del-expressing cells to that seen after HS. We observed a 1.5- and 2-fold increase in I-Hsp40 and I-Hsp70, respectively, in F508del-expressing CFBE cells relative to that seen in WT-expressing cells, whereas a 3.5- and 4-fold increase in I-Hsp40 and I-Hsp70, respectively, was observed after acute HS (Figure 1K). Thus, the level of HSR activation seen in response to chronic expression of F508del-CFTR represents approximately 50% of that seen during acute HS, indicating the presence of a subacute, chronic activation of the HSR pathway.

The transcriptional changes reported to occur in response to HSR activation [28]–[30] are likely to have a global impact on cellular function. Thus, we monitored the folding of firefly luciferase (FLuc), a sensor of folding stress in the cytosol [34],[45] that has also been used to monitor both ER and oxidative stress [46]–[50]. Here, we used the FLuc reporter not as an absolute measure of protein folding, but as a sensor for relative cytoplasmic folding stress when comparing control with diseased cells. Importantly, F508del-expressing cells exhibited a 50% reduction in the specific activity of FLuc compared to WT-expressing cells, which was restored to WT-like levels in response to F508del silencing (Figure 1L). Since the chronic activation of the HSR, observed in response to the expression of a misfolded protein, negatively impacts the folding of other cellular proteins as reported by FLuc, a state which is likely to impact multiple cellular function(s) (Figure 1M), we refer to this altered PN environment as a maladaptive stress response (MSR).

### The Hsp90 Co-chaperone p23 is a Key Regulator of the MSR

**Figure 2:**
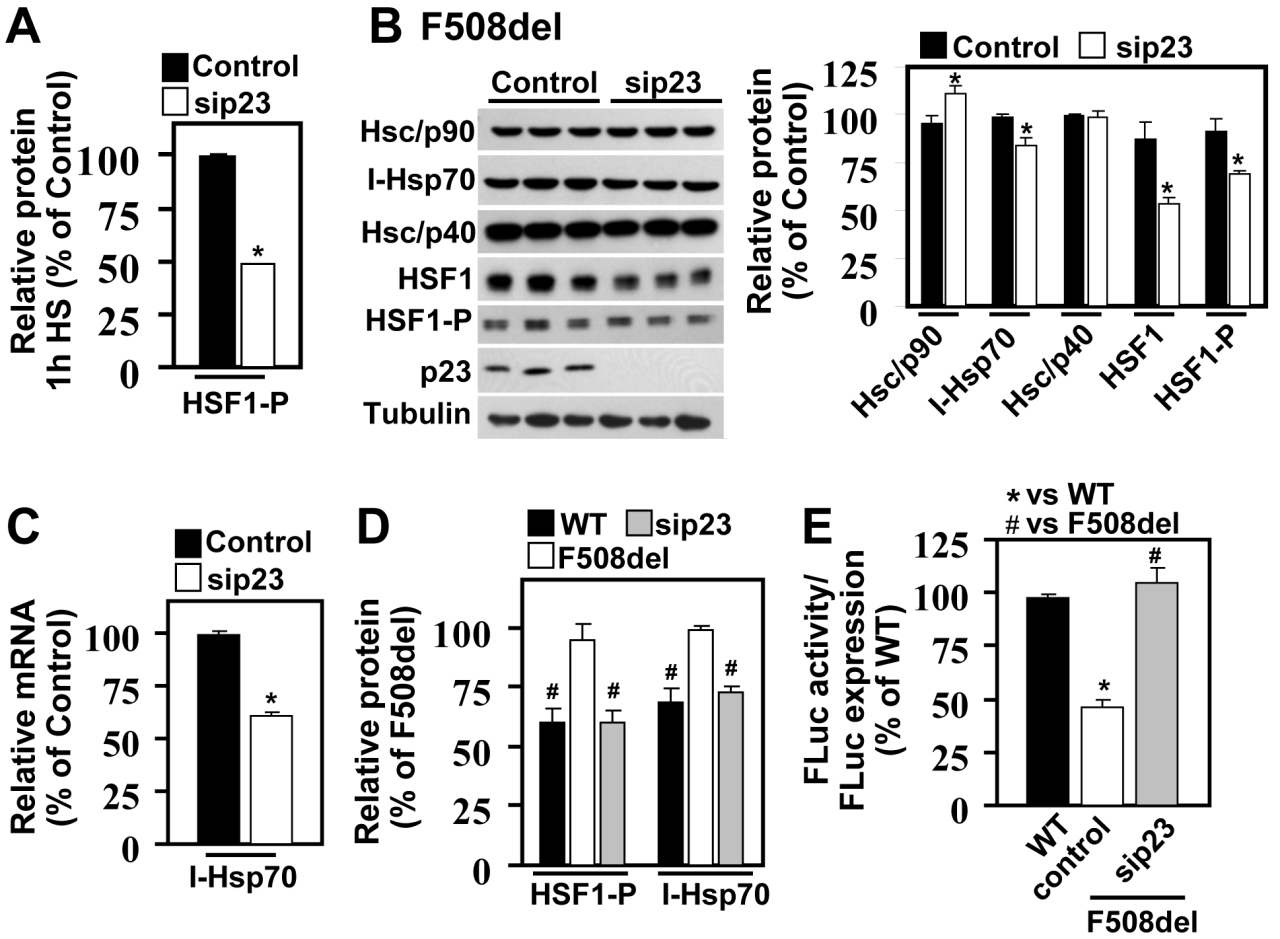
p23 Hsp90 co-chaperone is a modulator of the MSR. (A) Quantification of HSF1-P after 1 h HS in F508del-expressing cells treated with control or p23 siRNA. Data is shown as percentage of control (mean ± SEM, n = 4). (B) Immunoblot of the indicated proteins following p23 silencing in F508del-CFTR expressing cells. Histograms represent quantification of the indicated proteins upon sip23, relative to levels seen with control siRNA, which is set to 100%. (C) qRT-PCR analysis of I-Hsp70 in F508del-expressing cells following sip23. The data represent a ratio of I-Hsp70 to the housekeeping gene (GUS) and are shown as percentage of control siRNA. All results are shown (B,C) as a mean ± SEM, n≥3, and * indicates *p*<0.05 relative to control. (D) Quantification of HSF-1-P and I-Hsp70 protein levels in WT- and F508del- expressing cells after control or sip23 treatment. The data is shown as a percentage of F508del-expressing cells, and # represents *p*<0.05 relative to F508del (mean ± SEM, n≥3). (E) Quantitative analysis of FLuc activity in WT- and F508del-CFTR expressing cells treated with control or sip23. The data represents normalized specific activity of FLuc (luminescence/relative FLuc expression) for each condition (mean ± SEM, n≥3). All results were replicated at least once. The symbols * and # represent *p*<0.05 relative to WT and F508del respectively. The underlying data used to make (A–E) in this figure can be found in the supplementary file Data S1.

Given the increased activation of HSF1 in cells expressing F508del-CFTR, we next examined the impact of the Hsp90 co-chaperone, p23, an important regulator of HSF1 activity [8],[51],[52]. Since the MSR is a chronic response, we performed all small interfering RNA (siRNA) interventions for a total of six days to allow for appropriate rebalancing of the PN environment. P23 silencing significantly reduced HSF1 activation in response to HS, as exemplified by a reduction in the level of HSF1-P (Figure 2A), confirming its central role in the activation cycle of HSF1. At physiological temperature, p23 silencing in F508del-expressing CFBEs also resulted in a significant decrease in HSF1 and HSF1-P protein levels, as well as I-Hsp70 mRNA and protein levels (Figure 2B, 2C), to a level similar to that seen in WT-expressing cells (Figure 2D). Furthermore, abrogation of the MSR following p23 silencing led to a concomitant restoration of FLuc folding in F508del-expressing CFBEs (Figure 2E).

Silencing of p23 had no effect on HSF1 mRNA level (Figure S2A) nor on HSF1 stability, determined by pulse-chase (Figure S2B, S2C). However, we did observe a reduction in the amount of labeled HSF1 in the pulse-phase (Figure S2B, S2D), indicating a reduction in HSF1 translation in response to p23 silencing. In contrast, p23 silencing had no impact on HSF1 levels in WT-CFTR expressing cells, in which no MSR is detected (Figure S2E), suggesting that p23 plays a critical role in modulating the MSR induced in F508del-CFTR expressing cells.

### Modulation of the MSR by sip23 Improves F508del Channel Activity

**Figure 3:**
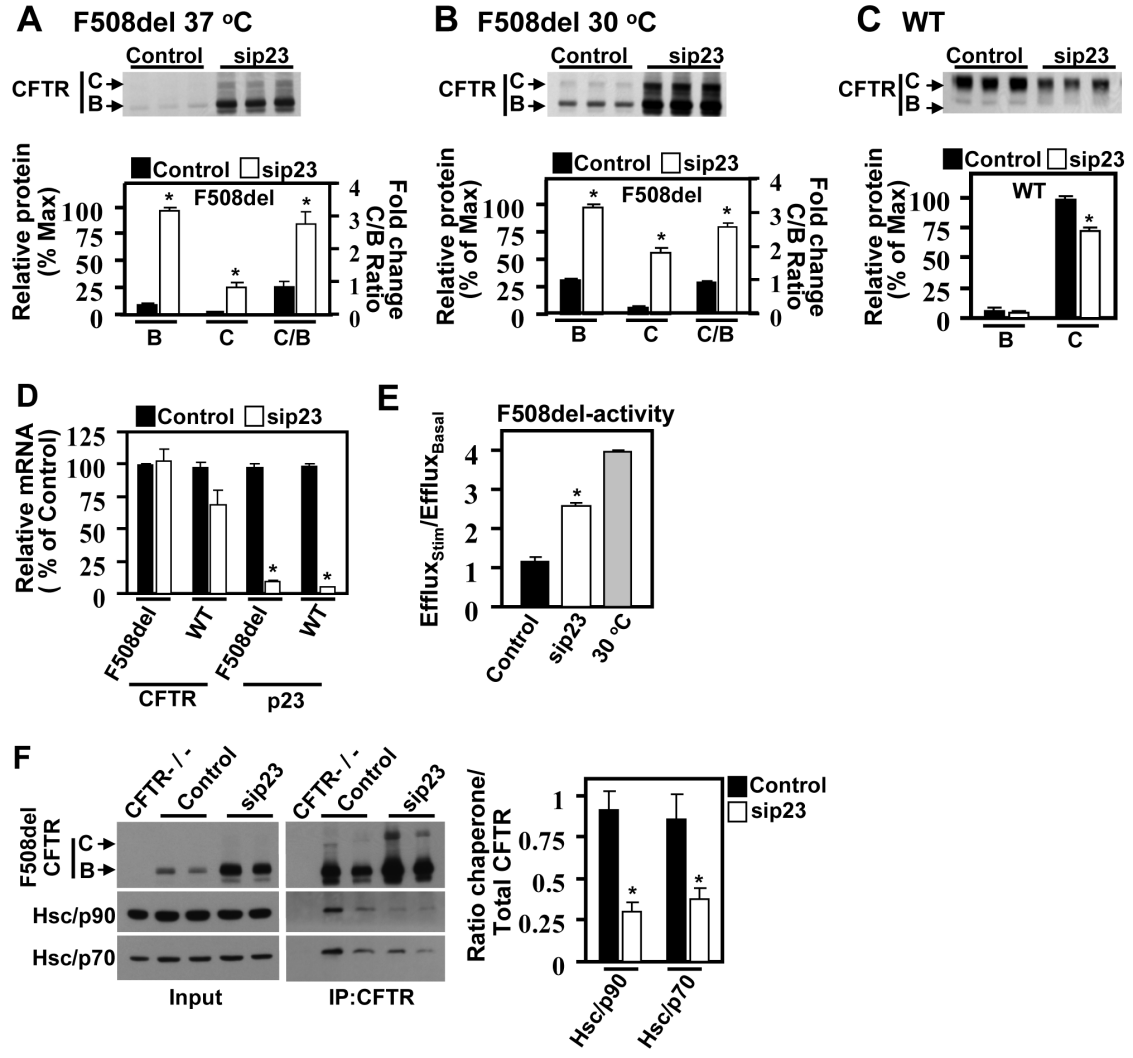
Silencing of p23 improves F508del-CFTR function in CF by down-regulation of the MSR. Immunoblot of CFTR following sip23 treatment of F508del-expressing cells at 37°C (A) and 30°C (B) or WT-expressing cells (C). Histograms show quantification of CFTR band-B and C glycoforms and C/B ratios. Results are shown as a percent of the maximal signal for band-B glycoform and as fold change relative to control (set to 1) for the ratio C/B (mean ± SEM, n≥3). (D) qRT-PCR analysis of CFTR and p23 levels following p23 silencing in WT or F508del-expressing cells. Results represent a ratio of the indicated mRNA to GUS, and shown as the percent of siRNA control (mean ± SEM, n≥3). (E) Iodide efflux analysis of F508del-expressing cells in response to p23 siRNA or 30°C correction. Results are shown as a ratio of the efflux at stimulation (stim) to efflux at pre-stimulation (basal) (mean ± SD, n≥3). (F) Immunoblot of the indicated proteins in the cell lysate (input) or following CFTR IP (right panels) in response to sip23 treatment (CFTR−/− cells were used as a negative control for the CFTR IP). Histogram shows quantification of recovered Hsc/p90 (α and β) and Hsc/p70 by co-IP with CFTR. The data is shown as a ratio of the recovered chaperone to total CFTR and normalized to 1 for the control siRNA (mean ± SD, n = 3, replicated three times). For all data, * indicates *p*<0.05 relative to control, and results were replicated at least once. The underlying data used to make (A–F) in this figure can be found in the supplementary file Data S1.

Since p23 silencing reduced the MSR state in F508del-expressing cells, we assessed its effect on F508del-CFTR biogenesis. P23 silencing resulted in a significant increase in F508del ER stability (band-B) and trafficking (band-C) compared to control siRNA treatment (Figure 3A). It also resulted in an increase in the trafficking index, defined as the ratio of band-C to band-B (C/B) [53], an indicator of its post-ER stability (Figure 3A). These results suggest that a reduction of the MSR, which restores a WT-like PN state (Figure 2D), supports the increased trafficking efficiency of F508del similar to what is observed following 30°C correction (Figure 3B), providing significant benefit to the CF phenotype. P23 silencing did not increase WT stabilization or trafficking (Figure 3C), indicating that its effect on F508del correction occurs in response to alleviation of the MSR exclusively seen in F508del-expressing cells.

CFTR pulse labeling in response to sip23 revealed a significant increase in the synthesis of F508del-CFTR but not of WT-CFTR (Figure S3B, S3D), consistent with the results presented above for the steady-state levels of CFTR (Figure 3A, 3C). This differential synthesis could be due to change in transcription, translation and/or post-translational stability of F508del-CFTR. Although p23 down-regulates the transcription of the glucocorticoid and thyroid hormone receptors [54],[55], we did not observe any changes in WT- or F508del-CFTR mRNA levels (Figure 3D). However, p23 silencing did significantly reduce the degradation rate of F508del-CFTR but not that of WT-CFTR (Figure S3A, S3C), suggesting that p23 specifically affects the stability of nascent F508del-CFTR. Increased F508del stability was not due to altered proteasome activity, since combining sip23 with the proteasome inhibitor, MG132, resulted in an additive effect on F508del stability and trafficking (Figure S3E). In support of this conclusion, the levels of ubiquitinated F508del following sip23 also remained unchanged (Figure S3F). p23 silencing also promoted a significant reduction of Hsc/p70 and Hsp90 (Hsp90α and Hsp90β) levels recovered in F508del-CFTR immunoprecipitates (Figure 3F), indicating that abrogation of the MSR allows F508del-CFTR to properly navigate early folding intermediates known to contribute to the ER retention of F508del-CFTR [42].

**Figure 4:**
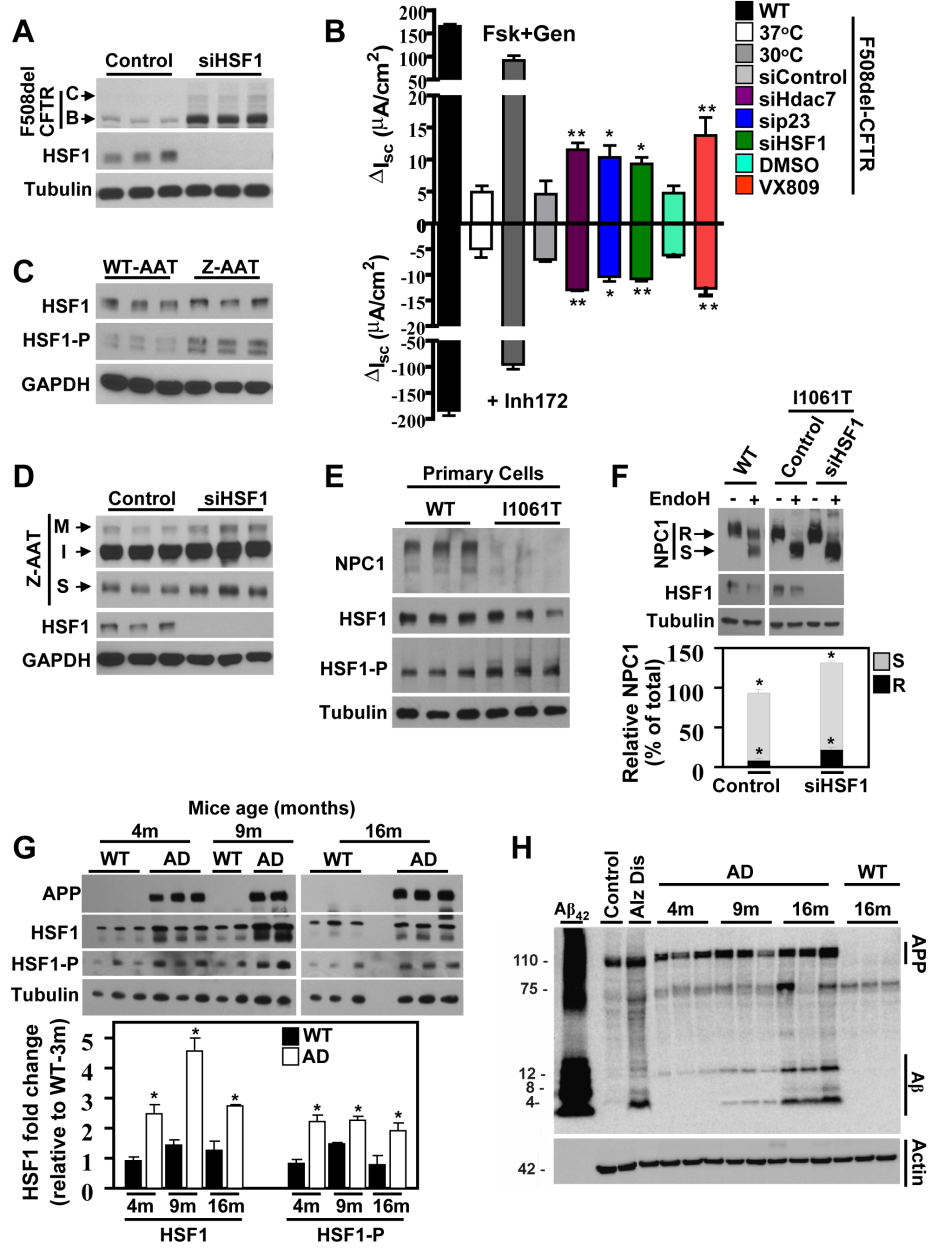
HSF1 silencing increases F508del folding, trafficking, and function and improves the phenotype of other misfolding diseases. (A) Immunoblot of indicated proteins following siHSF-1. (B) Short-circuit current (*I*_sc_) analysis of CFTR in polarized CFBE cells expressing WT- or F508del-CFTR treated with the indicated siRNA or temperature corrected at 30°C. Channel activity was determined in response to forskolin and genistein (positive deflection) or with CF specific inhibitor 172 (Inh172) (negative deflection). The ** indicates p<0.05 and * indicates p<0.1 relative to control siRNA or DMSO for VX809 (*n*≥4). (C) Immunoblot of the indicated proteins in WT- and Z-AAT expressing IB3 cells (*n* = 3). (D) Immunoblot of the indicated proteins in Z-AAT expressing IB3 cells in response to siHSF1 treatment. Shown for the AAT are the immature (I), mature (M), and secreted (S) forms (*n* = 3). (E) Immunoblot of the indicated proteins from primary fibroblasts derived from WT- or mutant I1061T-NPC1 patients (*n* = 3). (F) Immunoblot of the digestion pattern before and after endo-H digestion of WT- or mutant I1061T-NPC1 stably expressed in Hela cells and treated with the indicated siRNA (*n* = 3). Immunoblot shows NPC1 endo-H resistant band (R) and sensitive band (S). Quantification represents total I1061T NPC1 (R+S) in control or siHSF1-treated cells, shown as percentage of control. Percentage of endo-H sensitive band (S) or resistant band (R) is shown in gray and black color, respectively (*n*≥3, * indicates *p*<0.05). (G) Immunoblots of indicated proteins and quantification of HSF1 and HSF1-P on brain homogenates obtained from WT or AD mice of approximately 4 months (4 m; *n* = 3 for WT, *n* = 3 for AD), 9 months (9 m; *n* = 2 for WT, *n* = 2 for AD), and 16 months of age (16 m; *n* = 3 for WT, *n* = 3 for AD). Results were normalized to tubulin loading control and shown as fold change relative to WT-3m set to 1 (* indicates *p*<0.05 relative to age-matched WT mice). (H) Immunoblot for APP (110 kDa), Aβ_42_ toxic species (monomer, 4 kDa, and multimers from 6–12 kDa), and actin control on particulate fractions of brain homogenates obtained from WT (on the right) or AD mice. Controls shown include 0.5 µg recombinant Aβ_42_; control and Alzheimer disease samples from human brain homogenates. The underlying data used to make (B), (F) and (G) in this figure can be found in the supplementary file Data S1.

Given the observed correction of the F508del-CFTR trafficking defect by p23 silencing, we assessed whether the corrected pool of F508del was functional. F508del-expressing cells treated with sip23 exhibited a significant increase in channel activity, as determined by iodide efflux (Figure 3E) and short circuit current (I_sc_) recordings (see below, Figure 4B). Our results show that abrogation of the MSR by p23 silencing promotes trafficking of a functional F508del-CFTR to the cell surface.

### MSR Poses a General Challenge to Misfolding Diseases

Since the expression of F508del-CFTR results in chronic activation of HSF1, which not only affects F508del biogenesis but also the activity/folding of other cellular proteins (Figure 1L), we tested whether HSF1 silencing would also correct the trafficking defect associated with F508del-CFTR. HSF1 silencing resulted in a significant increase in ER stability (band-B), maturation (band-C) and trafficking index for F508del-CFTR (Figure 4A and Figure S4A). Furthermore, it also led to increase in F508del function by I_sc_ recordings to the level seen with siHDAC7, a validated siRNA target for correction of CF [56], and with VX809, a CF corrector currently in clinical trials for the treatment of F508del homozygous patients (Figure 4B) [57],[58].

In order to determine whether the MSR observed in CF is a general phenomenon associated with protein misfolding diseases, we monitored the HSR activation state in models of AATD, NPC1, and AD. In AATD, the G342K mutation in AAT, referred to as the Z-variant, results in ER-retention, polymerization, and degradation of this normally secreted enzyme, the loss of which leads to COPD [32],[59]. Cells expressing the Z-variant exhibited higher levels of HSF1-P compared to WT-AAT expressing cells (Figure 4C and Figure S4B), suggesting, once again, the existence of a MSR. Furthermore, HSF1 silencing resulted in increased maturation (AAT-M) and secretion (AAT-S) of the mutant Z-AAT (Figure 4D and Figure S4C, S4E); however, no changes in the polymerization state of the Z-AAT variant were observed (Figure S4D). This result is consistent with the effect of other correctors, such as suberoylanilide hydroxamic acid (SAHA), in which increased maturation and secretion of Z-AAT is observed without changes in polymerization [33]. Thus, MSR abrogation also provides benefit to a protein misfolding disease found in the ER lumen [9],[33], suggesting a link between ER stress biology [15] and cytoplasmic stress management by HSR. This observation is consistent with previous results suggesting a crosstalk between these pathways [60]. In addition, genomic analysis has revealed that transcriptional targets of HSF1 found in the secretory pathway are also induced by UPR [61]–[63], providing a mechanism by which silencing of HSF1 could be beneficial for AATD.

We next investigated whether a MSR arose in response to the I1061T variant of the NPC1 protein responsible for the lysosomal storage disease Niemann-Pick type C1, which, like CF, is characterized by protein misfolding and ERAD-mediated clearance [64]. An analysis of human primary fibroblasts from homozygous I1061T NPC1 patients and healthy donors (WT) reveals that cells expressing the I1061T variant exhibit an elevation in the levels of HSF1-P, suggesting the presence of a MSR (Figure 4E and Figure S4F). Here HSF1 silencing also improved the trafficking defect of I1061T-NPC1, as exemplified by the increased endo H resistance reflecting modification of its N-linked oligosaccharides by Golgi enzymes, relative to that seen with control siRNA (Figure 4F).

We then examined a *Caenorhabditis elegans* model of cytoplasmic amyloid aggregation. *C. elegans* expressing the β-amyloid-42 (Aβ_42_) peptide fused to CFP (Aβ_42_-CFP) under the control of a muscle-specific unc-54 promoter forms CFP-positive Aβ aggregates in the cytoplasm of muscle cells (Figure S5A, S5B). The *C. elegans* model has been extensively used in the field of misfolding diseases and is a validated tool to study the impact of amyloid disease in organismal models [19],[21],[65],[66]. Here we observed an increase in I-Hsp70 level in Aβ_42_ worms (∼150-fold, Figure S5C), which was not further up-regulated after HS as seen in WT worms. Up-regulation of I-Hsp70 was reduced in response to HSF1 silencing or reduction of Aβ_42_ expression (Figure S5D), indicating that the misfolding stress caused by Aβ_42_ expression also induces a MSR state. Accumulation of cytosolic Aβ_42_ aggregates led to paralysis in 75% of diseased worms relative to its WT counterparts, which was significantly reduced by silencing of not only Aβ_42_ (silencing of yellow fluorescent protein- [siYFP]) but also in response to I-Hsp70 and HSF1 silencing (Figure S5E, S5F). Conversely, HSF1 overexpression resulted in increased Aβ_42_ induced proteotoxicity with an approximately 30% increase in paralyzed worms (Figure S5G).

To extend these observations to a neurodegenerative model of Aβ_42_ amyloid aggregation, we examined the expression levels of HSF1 and HSF1-P (phosphorylated at T^142^) [67] in brain homogenates of WT and AD mice (AβPP Tg) at three different ages (approximately 4 mo, 9 mo, and 16 mo old). We observed a significant increase in both HSF1 and HSF1-P expression in all AD mice compared to their age-matched WT counterparts (Figure 4G). The toxic Aβ_42_ amyloid species (4 kDa monomer and 6-12 kDa multimers) [68],[69], previously characterized in this AβPP Tg mice model [70], were detected in brain homogenates from AD mice but not in that of WT mice. The accumulation of Aβ_42_ amyloid in AD mice was also age dependent (Figure 4H), consistent with previously published studies showing age-dependent increase in Aβ plaques, and mean plaque size on these mice [70]. Despite the age-related increase in toxic amyloid, we did not observe an age-dependent increase in HSF1-P in the AD mice, a result consistent with the known decline of proteostatic capacity as has been previously documented in aging organisms in the face of increasing cellular stress [71]–[73].

### Silencing of HSF1 Improves F508del Folding and Its Cell Surface Stability

**Figure 5:**
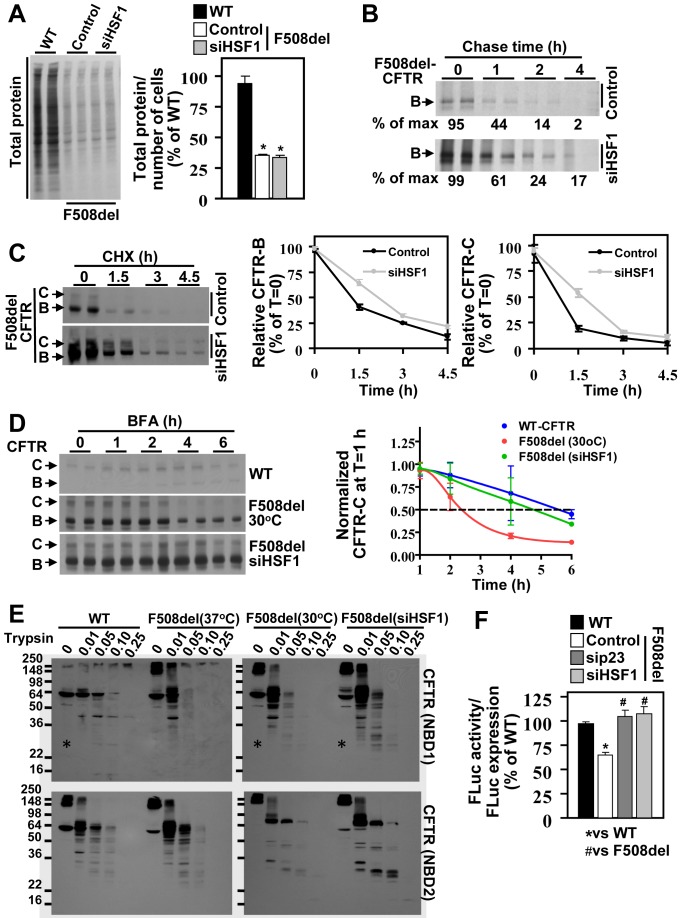
Silencing of HSF1 improves F508del folding and its cell surface stability. (A) Total protein synthesis on S^35^ labeled samples from WT- or F508del-CFTR expressing cells treated with control or siHSF1. Results are shown as percent of WT for total protein normalized by number of cells in each condition (mean ± SEM, *n* = 3; * indicates *p*<0.05 relative to WT). (B) Pulse-chase of F508del-CFTR in response to siHSF1 treatment. Numbers show percent of F508del relative to time 0 (T = 0; *n* = 2). (C) F508del-CFTR immunoblots and quantification in control or siHSF1 transfected cells before (T = 0) and after cycloheximide (CHX; 50 µM) chase for the indicated time (h). Results are shown as percent of band-B or band-C at T = 0 (mean ± SD, *n*≥4). (D) CFTR immunoblots and quantification in WT or F508del corrected at 30°C or by siHSF1 before (T = 0) and after brefeldin A treatment (BFA; 5 µg/ml) for the indicated time (h). The data is presented as a fraction of maximal band-C set to 1 at 1 h post-BFA treatment and represents the mean ± SD, *n*≥4. (E) Representative immunoblots from three experimental replicates of CFTR—WT, F508del at 37°C, F508del at 30°C, or F508del siHSF1—before and after proteolysis digestion with increasing concentration of trypsin (mg/ml). Upper blots show digestion pattern of CFTR NBD1 domain (18D1 antibody), and lower blots show digestion pattern of CFTR NBD2 domain (M3A7 antibody) (* indicates stable core fragment previously described) [75]. (F) Quantification of FLuc activity in WT- and F508del-CFTR expressing cells treated with the indicated siRNA. The data represents specific FLuc activity (luminescence/relative FLuc expression) for each condition. Results are shown as a mean ± SEM, *n*≥3; * and # indicate *p*<0.05 relative to WT- and F508del-CFTR, respectively. The underlying data used to make (A), (C), (D) and (F) in this figure can be found in the supplementary file Data S1.

The MSR is a chronic state transferring the misfolding challenges to all aspects of cellular folding biology managed by proteostasis components impacting the activity of the Q-state of F508del [42]. Thus, we examined in more detail the impact of HSF1 silencing, which in our CF cell model resulted in increased stability and trafficking of F508del-CFTR at steady state (Figure 4A). To address whether the observed increased in F508del stability reflected an increase in global protein synthesis, we compared the level of S^35^-labeled proteins in cellular lysates from F508del-expressing cells in the presence or absence of siHSF1 to that seen in WT-expressing cells. Strikingly, we first observed that MSR-affected F508del-expressing cells exhibited a drastic decrease in total protein synthesis, representing less than 50% of that seen in healthy WT-expressing cells (Figure 5A). This highlights the negative impact of MSR activation on the proteome and is consistent with attenuation of protein synthesis seen in numerous types of stress [74]. HSF1 silencing had no impact on the level of total protein synthesized in F508del-expressing cells (Figure 5A); however, we did observe an increase in F508del synthesis after pulse labeling, followed by increased stability of de novo synthesized F508del band-B in the chase phase of the experiment (Figure 5B). We also observed increased stability of band-C after inhibition of de novo protein synthesis by cycloheximide (CHX) treatment (Figure 5C). To determine if band-C stability resulted from increased band B to C trafficking following CHX treatment, we used brefeldin A (BFA) to block ER to the Golgi trafficking and track the stability of rescued F508del-CFTR (rF508del) band-C by preventing egress to the cell surface. The half-life (T1/2) of band-C in temperature-rescued F508del (rF508del) was approximately 2 h, whereas HSF1 silencing significantly increased the stability of the rF508del pool, exhibiting a T1/2 of 6 h, a value similar to that seen for WT-CFTR (Figure 5D). These data suggest that alteration of the MSR by siHSF1 increases the stability of rF508del band-C, possibly as a result of improved protein folding.

To directly address whether we have achieved improved protein folding following siHSF1, we used limited trypsin proteolysis, a method previously shown to distinguish between the stable and destabilized fold of the WT and F508del variants, respectively [75]. We used antibodies specific for the first nucleotide binding domain (NBD1: 18D1) and second nucleotide binding domain (NBD2: M3A7) of CFTR, to assess the susceptibility of these domains to resist proteolysis. HSF1 silencing leads to a significant stabilization of both NBD1 and NBD2, exhibiting a more pronounced stabilizing effect to that seen with temperature correction (Figure 5E). It also led to the appearance of an approximately 25 kDa band in NBD1, which has previously been described to represent the stable core fragment seen in WT-CFTR, but not in the F508del variant [75]. HSF1 silencing also restored the folding of the FLuc reporter to a level comparable to that seen in sip23-treated F508del-expressing cells and WT-expressing cells (Figure 5F). Overall our results suggest that alleviation of the MSR by siHSF1 generates a more permissive cellular environment for productive folding, not only improving the CF phenotype but also that of other protein misfolding diseases by restoring a WT-like proteostasis environment.

To understand the impact of HSF1 silencing on F508del-CFTR stability, we performed gene expression analysis. Here we found that HSF1 or p23 silencing leads to a reduction in the expression of HSF1-responsive genes, such as I-Hsp70, HSPB1, and I-Hsp40. However, they had no effect on the transcription of CFTR itself (Figure S6A), nor the expression levels of markers for other PN cellular pathways, including ubiquitin proteasomal system (UPS), autophagy, and oxidative stress (NRF2 pathway) (Figure S6B). In addition to the alleviation of the HSR, both siHSF1 and sip23 also decreased the expression of UPR-related genes (Figure S6B). UPR but not the oxidative stress pathway was up-regulated in F508del-expressing cells in comparison with WT-expressing or CFTR−/− cells (Figure S6C), suggesting a link between HSR and UPR activation, as previously described [60].

Finally, we used the proteasomal inhibitor MG132 and the autophagy inhibitor 3-methyladenine (3-MA) to examine the impact of proteasome and autophagic pathways on the FLuc folding sensor. Whereas HS of F508del-expressing cells further reduced FLuc activity and folding from a level of 50% to 25% of that of WT-cells (Figure S6D), neither MG132 nor 3-MA impacted FLuc folding in F508del-expressing cells. These results suggest that blocking proteasomal activity or autophagy is not sufficient to rescue FLuc folding in an environment already affected by the MSR.

### Chemical Inhibition of HSF1 Activation Promotes Correction of CF

**Figure 6:**
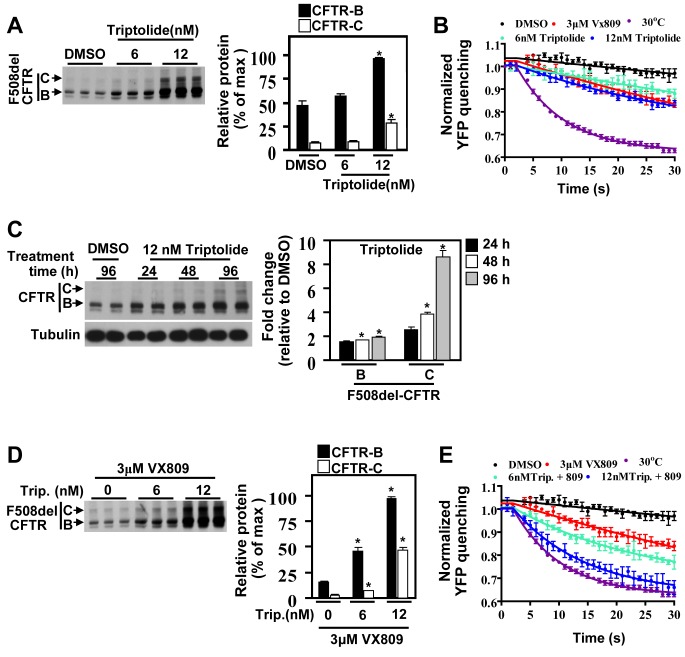
Chemical inhibition of HSF1 improves F508del-CFTR function. Immunoblot and quantification of CFTR following treatment of F508del-CFTR–expressing CFBE cells with increasing concentration of triptolide (Trip.) alone (A) or in combination with the CF corrector VX809 (D). Results are expressed as percentage of maximum signal of CFTR band-B (set at 100%), and shown as mean ± SEM, *n*≥3; * represents *p*<0.05 relative to DMSO. (C) Immunoblot and quantitative analysis of CFTR following a daily chronic dosing regimen (96 h) of 12 nM triptolide in F508del-CFTR expressing cells. Results are expressed as fold change relative to DMSO, and shown as mean ± SD, *n* = 2; * represents *p*<0.05 relative to DMSO. YFP-quenching curves of F508del-CFTR expressing CFBE-YFP cells treated with the indicated compounds for 24 h alone (B) or in combination (E) (mean ± SD, *n*≥3). All results were repeated at least once. The underlying data used to make (A–E) in this figure can be found in the supplementary file Data S1.

Given the impact of the MSR on the recovery of F508del function, we tested the effect of chemical inhibition of HSF1 in F508del-expressing CFBEs, using the previously characterized HSF1 inhibitor, triptolide [76]. Triptolide reduced the HS-induced up-regulation of I-Hsp70 and I-Hsp90 mRNA levels (Figure S7A), confirming its ability to block HSF1 transactivation, consistent with previously published data [76]. Treatment of F508del-expressing cells with triptolide resulted in an increase in band-B stability as well as trafficking to band-C (Figure 6A). It also restored cell surface channel activity shown by quenching of the halide sensing YFP-H148Q/I152L (Figure 6B), to a level similar to that seen with VX809 (Figure 6B). Since misfolding diseases present a chronic challenge to the cell, we next assessed the benefit a chronic dosing regimen of triptolide on correcting the F508del-CFTR trafficking defect. Chronic treatment resulted in a time-dependent increase in stabilization and trafficking of F508del-CFTR over the course of 96 h (Figure 6C). The effect of triptolide was dependent on HSF1, since combining triptolide and siHSF1 did not result in additivity for F508del stability, trafficking, and function (Figure S7B), further supporting the conclusion that suppression of HSF1 hyper-activation promotes F508del correction.

Since down-regulation of the MSR provides a favorable environment for protein folding and trafficking of F508del-CFTR, we re-assessed the potency of existing correctors of F508del-CFTR in combination with triptolide or siHSF1. Treatment of F508del-expressing cells with VX809 or triptolide alone led to a moderate restoration of F508del-CFTR activity (Figure 6B). In contrast, combining both drugs had a synergistic effect on F508del-CFTR trafficking and channel activity (Figure 6D, 6E and Figure S7C). Similar results were also observed with siHSF1 in combination with VX809 and other CF correctors (Figure S7D), showing that alleviation of the chronic stress improves the potency of clinically relevant correctors of F508del trafficking and function.

### Abrogation of the MSR Increases F508del Function in CF Patient-Derived Bronchial Epithelia and Intestinal Organoids

**Figure 7:**
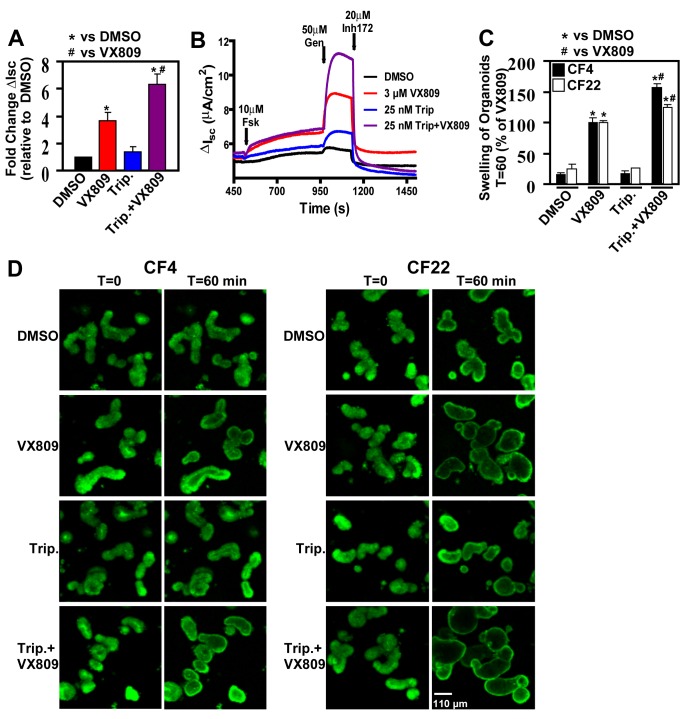
Chemical inhibition of HSF1 synergizes with VX809 to improve F508del-CFTR function in patient-derived primary epithelium. (A) Short-circuit current analysis of human primary hBE cells (F508del/F508del, patient code CF006) treated with DMSO, 3 µM VX809, and 25 nM triptolide or a combination of VX809 and triptolide, for 96 h (daily dosing). The data is presented as fold change relative to the basal current seen with DMSO treatment, and shown as mean ± SD, *n*≥3 (replicated multiple times); * and # indicate *p*<0.05 relative to DMSO or VX809, respectively. (B) Representative short-circuit current (I_sc_) traces for DMSO, VX809, triptolide, or triptolide + VX809 treatment of primary hBE cells from (A). (C) Quantitative analysis of organoid swelling (shown in D) that is indicative of CFTR function over the period of 60 min. Organoids were obtained from two distinct F508del/F508del CF patients (CF4, CF22), and treated with DMSO, 3 µM VX809, 25 nM triptolide, or a combination of VX809 and triptolide. Experiments were repeated once and results are shown as a mean ± SD, *n*≥2; * and # indicate *p*<0.05 relative to DMSO or VX809, respectively. (D) Representative images of organoids derived from patients (CF4 and CF22) at T = 0 or after stimulus with Forskolin/Genistein at T = 60 min treated with the indicated compounds. Scale bar represents 110 µm. The underlying data used to make (A–C) in this figure can be found in the supplementary file Data S1.

We next examined the effect of triptolide treatment in patient-derived bronchial hBE cells homozygous for F508del. Treatment with triptolide resulted in a modest 1.4-fold increase in short-circuit current (I_sc_) relative to that seen with vehicle treatment (Figure 7A). Maximal correction was obtained when triptolide was combined with VX809, resulting in a 7-fold increase in I_sc_ over the basal current (Figure 7A, 7B), synergizing with the VX809 effect, which achieved a 3.5-fold increase in I_sc_. To address whether this effect was tissue specific, we also tested the effect of triptolide using primary CF intestinal organoids derived from two F508del CF patients [77]. In this assay, increased organoid swelling is indicative of restored F508del function. Although we did not observe any effect with triptolide alone, we did observe an approximate 50% increase in organoid swelling when VX809 was combined with triptolide as compared to that seen with VX809 alone, revealing a synergistic response in both CF patient codes (Figure 7C, 7D), similar to that seen in primary hBE and CFBE cells. These results highlight the beneficial impact of MSR abrogation and its ability to improve the potency of existing therapeutics, consistent with our hypothesis that restoration of a WT-like folding environment could be a critical factor in managing human misfolding disease [5],[9].

## Discussion

**Figure 8:**
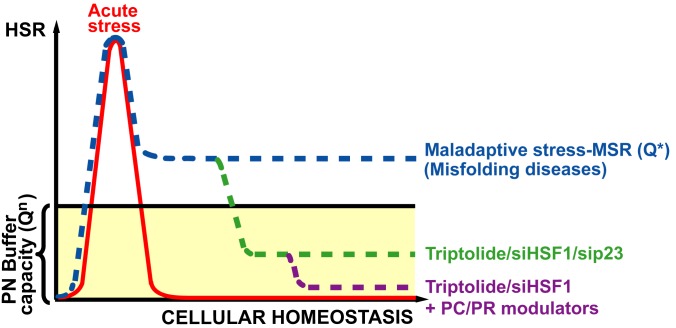
Q-state management of MSR to correct human disease. Illustrated is the activation state of the HSR in response to acute stress (red) or to the MSR (blue) seen in disease. Acute HSR activation, seen during acute stress insults, protects from and/or corrects misfolding and rapidly returns to basal levels, allowing normal biology to resume. In misfolding disease, chronic activation of the HSR alters the normal, physiologic Q-state (Q^n^) because of the continued expression of misfolded protein. Once chronically elevated (Q*), the folding environment becomes maladaptive as it fails to return to the Q^n^ (light yellow area). Down-regulation of the MSR by siHSF1, sip23, or triptolide promotes a reduction of the Q*, which now falls within the proteostasis buffering capacity (green line), promoting a more normal cellular folding environment. This effect can be further improved (purple line) when combined with protein fold correctors (pharmacologic chaperones; PCs) which impart improved thermodynamic stability to the fold, or proteostasis regulators (PRs) that improve protein Q-state biology, improving function of disease-related misfolded protein and its proteome's associated environment, promoting abrogation of the chronic stress and improving health.

Our results demonstrate that the long-term expression of disease-causing misfolded proteins can lead to an abnormal, chronic stress response that we now refer to as the maladaptive stress response (MSR). This altered Q-state [3]–[6], which emphasizes that the structure of a protein is tightly integrated with a dynamic proteostatic system [1],[5],[6],[9], negatively impacts the folding of disease-associated proteins, such as F508del-CFTR [42], leading to a self-propagating proteotoxic crisis (Figure 8). We have found that targeting the MSR can significantly alleviate disease progression, thereby improving the disease phenotype in different disease models of protein folding. In CF, this is consistent with the view that folding of CFTR is a multi-step, vectorial process involving sequential folding intermediates that must be therapeutically managed for effective correction [42],[78],[79]. We now suggest that restoration of the native cellular proteostasis-state could represent a critical first line of therapeutic intervention to more effectively achieve the correct structure–function relationship necessary to restore cellular function.

Our results show that the proteostatic biology of F508del-expressing cells is different than that seen in WT-expressing cells, characterized by a subacute increase in heat shock protein expression, reduced protein synthesis, and altered protein folding, phenomena contributing to the disease phenotype that we have referred to in the past as the chaperone trap [42]. These results are consistent with previous observations where elevated levels of heat shock proteins were observed in postmortem brain tissue of AD patients [80]–[83], and in lung tissue of COPD patients [84]. Our proposed paradigm shift in how to address protein misfolding diseases leads us to suggest that, unlike the well-documented protective benefit of HSR activation to solve acute and transient protein misfolding problems (see below) [40], the MSR is counterproductive when chronically activated, attempting to repeatedly manage a misfolding problem that it cannot solve. This condition thereby exacerbates the disease rather than relieving it, emphasizing the importance of first managing the disease from the perspective of proteostasis by mitigating the chronic folding stress problem. We propose that abrogation of the MSR, either by directly stabilizing the initiating misfolding intermediate [34],[85],[86] or, as suggested herein, through restoration of a WT-like Q-state [5],[6],[9], could provide substantial benefit to counter the proteotoxic crisis found in chronic disease (Figure 8).

It is becoming increasingly evident that there exists a fine balance between protection and toxicity in the function of the protein folding environment in eukaryotic cells [2],[5],[6],[22],[87]. On one hand, the beneficial impact of HSR activation in preventing proteotoxicity in worm and mouse models of HD and AD [88]–[90] and in promoting cell survival in the face of diverse stress insults has been well documented [8],[9],[27]. Additionally, HSF1 activators and overexpression of select chaperones have been shown to be neuroprotective [36],[91]–[93]. However, the mechanism of action of such compounds and the chronic effect of HSF1 activation in vivo remain to be elucidated. Proteostasis regulators shown to activate HSF1 and to provide benefit in HD have also been shown to affect other stress pathways, including oxidative stress and UPR, which could contribute to disease management [36]. HSF1 overexpression has also been shown to exacerbate mutant Htt aggregation in a cellular model of HD [37]. On the other hand, Hsps are known to be actively involved in disease progression [80],[82],[83]. For example, in tau pathology, Hsp90 binding promotes tau misfolding and aggregation [94], not unlike the chaperone trap state found in CF [42],[78],[79], a result consistent with the dynamic state of the disordered tau protein and its interaction with Hsp90 in disease [95]. Moreover, chaperone balance is disrupted upon overexpression of polyQ aggregates through sequestration of low-level expression regulatory co-chaperones required for protein folding [96]. While Hsp90 inhibitors, which indirectly activate HSF1, show promise in treating neurodegenerative diseases [97],[98], the beneficial effect was shown to be directly due to Hsp90 inhibition, which, in the case of tauopathies, reduces the functional cycling of kinases and thereby tau phosphorylation, minimizing its aggregation and toxicity [99],[100]. Thus, while the mechanism of action of HSF1 activation is poorly understood, perhaps reflecting experimental conditions where a ‘brief’ burst of chaperones provides temporary relief to the misfolding problem, there is limited evidence in vivo that chronic activation of HSF1 provides long-term disease benefit. Indeed, proliferation of cancer cells is also dependent on a MSR characterized by sustained HSR activation and elevated levels of proteostatic components that sustain invasive survival [28],[38], a pathological condition leading to reduced human lifespan.

The global proteotoxic crises that arise in protein misfolding diseases may be a consequence of an amplifying cascade of misfolding challenges as disease progresses, a view consistent with reports of reduced longevity in worms following chronic overexpression of misfolded proteins [35],[90],[101],[102]. Alternatively, disease progression could reflect either the loss of proteostatic capacity associated with aging [4],[8],[21],[73],[103]–[105] or an overload of the cellular PN capacity. In the latter case, since Hsc/Hsp70 and Hsp90 represent at least 0.5% and 1% of total cellular protein, respectively, and cells exhibiting a MSR have reduced global protein synthesis, it is unlikely that the chaperone capacity per se is saturated, but this remains to be tested directly, given the complexity of the folding environment and lack of understanding of chaperone capacity in each cell type and/or disease environment. However, we have observed that the silencing of key proteostatic chaperones leads to a partial rescue of F508del-CFTR cell surface channel activity (Figure S7E) [106], arguing against a possible overload of the chaperone capacity, at least in CF disease. Indeed, the reduced specific activity of the FLuc sensor suggests a significant challenge to the overall cellular folding environment, a result that is consistent with the recent observation that overexpression of the Hsp40/70 system decreases the fraction of protein that achieves a functional fold using activity-based profiling [11]. These observations underline the importance in understanding folding mis-management by the chronic MSR that exceeds a set-point defined by chaperone/co-chaperone balance normally required for a healthy cell. It is clear that this new principle of short-term acute versus long-term chronic proteostatic set-points now needs to be considered as an important contributor to the onset and progression of misfolding diseases such as CF, AATD, NPC1, and AD. For example, the activity of FLuc, a sensor of the folding environment of the prevailing PN [45],[46]–[50], in cells chronically expressing the misfolded F508del-CFTR was reduced in response to elevated HSF1 activity, but restored to WT-levels upon MSR abrogation by siHSF1, sip23 or, importantly, following removal of the misfolded F508del-CFTR. Here, we suggest that p23, acting in concert with Hsp90 in protein folding and transcriptional activation of HSF1, accentuates the activity of the chaperone trap components, engaging F508del in an inappropriate attempt to resolve progression along the folding pathway [42]. Consistent with this conclusion, we observed HSF1 phosphorylation and I-Hsp70 levels, in response to sip23, reduced to the levels seen in WT-expressing cells, thereby restoring a WT-like PN that would be expected to be optimized for CFTR biogenesis and proteome function. While abrogation of the MSR by siHSF1 did not affect CFTR transcription, global protein synthesis, or other tested PN pathways (UPS, autophagy, and oxidative stress), it specifically abrogated both the HSR and UPR activation, restoring function. It also improved folding and activity of the FLuc reporter sensor. Thus, we now suggest that early translation-linked events could be critical determinants of HSR, disease onset and/or progression promoting the MSR, a conclusion consistent with the increasing regulatory complexity of the HSR at the level of transcription [96],[107],[108].

Why does the HSR work acutely but trigger a maladaptive state when chronically active in misfolding disease, triggering MSR? One possibility is that during evolution, the HSR pathway evolved strategies to manage long-term proteostasis states that are necessary for optimizing stemness [21],[105],[109] and/or direct long-term development, differentiation and multi-organ genesis, required for integrated organismal function, and to extend lifespan [24],[110]. Such a finely tuned Q-state in higher eukaryotes may be less permissive to fluctuations in PN biology in response to inherited variants in human disease that become out of reach of the normal proteostasis buffering capacity, and therefore more prone to maladaptation [5],[28],[39],[111]. Curiously, maladaptation not only includes the role of the HSF1-Hsp90 axis in supporting proliferation of cancer cells, a pathogenic state [28],[38],[39],[112], but also the propagation and resistance of viral pathogens to host defenses that can impact human health [113]–[115]. We would now propose maladaptation as a potent force in evolvability [21],[105],[109], contributing to improved survival and fitness [5],[6],[18],[116], highlighting an important principle applicable to correction and increased survival in response to chronic human disease, perhaps through epigenetic mechanisms that, we now appreciate, play a central role in HSF1 management [107],[108] and correction of human disease [28],[33],[56]. We now suggest that an appreciation of the impact of maladaptation on protein folding dynamics managed by the Q-state [4],[5] could provide insight into how to effectively manage the vast array of chronic protein misfolding states affecting human disease [1].

## Materials and Methods

### Cell Lines

Human bronchial epithelial cells CFBE41o- stably expressing F508del-CFTR or WT-CFTR were cultured as previously described [56]. IB3 cells expressing WT-AAT or Z-AAT were cultured as previously described [33]. For all temperature-corrected experiments, F508del-CFTR expressing CFBE cells were transferred to 30°C for 24 h. Hela cells stably expressing WT or I1061T-NPC1 were cultured in Dulbecco's Modified Eagle Medium (DMEM) containing 10% (v/v) fetal bovine serum (FBS), 2 mM L-glutamine, 3 µg/ml puromycin, and 600 µg/ml G418. Primary fibroblasts derived from healthy donors (WT) or patients homozygous for the I1061T mutation of NPC1 were cultured in DMEM containing 10% (v/v) FBS, and 2 mM L-glutamine.

### Culturing Primary Human Bronchial Epithelia Cells

Cells were obtained from Scott Randall at UNC Chapel Hill. Cells were plated on PureColl100 coated plates and grown in bronchial epithelial growth media (BEGM) + bullet kit (Lonza) + 1 µM all-trans retinoic acid with daily media changes until cells reached 90% confluence. Cells were harvested with Accutase at 37°C for 10 min and pelleted at 500 g for 5 min. Cells were re-suspended in BEGM and plated on human placental collagen coated 12 mm Costar snapwell filters (Corning) at a density of 5×10^5^ cells/filter. Cells were grown in liquid/liquid culture for the first 96 h with daily media changes as previously described [117] on the apical (0.5 ml) and basolateral (2 ml) chambers. Cells were subsequently switched to air/liquid culture and basolateral media, changed every day for the first 7 days and three times a week for 4–6 weeks until differentiation was complete.

### Culture of Organoids from Human Rectal Biopsies

Culture of organoids was performed as previously described [77]. Briefly, biopsies were washed with cold, complete chelation solution and incubated with 10 mM EDTA for 30 (small intestine) or 60 (rectum) min at 4°C. Crypts were isolated by centrifugation and embedded in Matrigel (growth factor reduced, phenol free; BD bioscience) and seeded (50–200 crypts per 50 µl Matrigel per well) in 24-well plates. The Matrigel was polymerized for 10 min at 37°C and immersed in complete medium (DMEM/F12 with penicillin and streptomycin, 10 mM HEPES, Glutamax, N2, B27 [Invitrogen], 1 µM N-acetylcysteine [Sigma]) and the following growth factors: 50 ng/ml mouse epidermal growth factor (mEGF), 50% Wnt3a-conditioned medium and 10% noggin-conditioned medium, 20% Rspo1-conditioned medium, 10 µM nicotinamide (Sigma), 10 nM gastrin (Sigma), 500 nM A83-01 (Tocris), and 10 µM SB202190 (Sigma). Medium was changed every 2–3 days. Organoids were passaged every 7–10 days, and passages 1–10 were used for confocal live-cell imaging.

### Generation of WT-CFTR and F508del-CFTR Stable CFBE Cell Lines Expressing WT Firefly Luciferase (FLuc)

The gene coding for the eYFP fluorescent protein was fused at the C-terminus of the WT Firefly luciferase gene (FLuc) and cloned into the lentivirus vector, pLVX-Puro (Clontech). CFBE cells stably expressing WT- or F508del-CFTR were infected with 5×10^6^ PFU of pLVX-Puro-eYFP-FLuc lentivirus. Cells expressing eYFP-FLuc fusion protein were sorted by FACS to generate WT- or F508del-CFTR CFBE cell lines stably expressing eYFP-FLuc.

### siRNA Knockdown, Overexpression, and Preparation of Cell Lysates and Western Blotting

siRNA transfections and preparation of cell lysates and Western blots was done as previously described [56]. For overexpression experiments, cells were plated at 70% confluency in a 12-well plate and transfected using 1 µg of DNA, 2 µl of P3000 per µg of DNA, and 1.5 µl of lipofectamine 3000 in Opti-MEM containing 5% FBS (Life Technologies). Cells were washed and fed on the next day and lysed 48 h after transfection.

### qRT-PCR

qRT-PCR was performed using the iScript One-Step RT-PCR kit with SYBR green (Bio-Rad). RNA was standardized by quantification of beta-glucuronidase (GUS) mRNA, and all values were expressed relative to GUS. Statistical analysis was performed on three independent technical replicates for each RNA sample, where error bars represent SD or SEM.

### Immunoprecipitation

For each immunoprecipitation (IP), 1 mg of total protein was used. CFTR IP was performed as previously described [118]. For HSF1 IP, cells were lysed in 20 mM Tris-HCl pH 7.4, 130 mM NaCl, 10 mM Na_2_MoO_4_, 1 mM EDTA, 5 µM ATP, 0.5% NP-40, and 2 mg/ml of complete protease inhibitor cocktail. Lysates were incubated with 3 µl of HSF1 antibody (Abcam, ab52757) for 18 h, and complexes were recovered with 30 µl of γ-bind beads incubated at 4°C for 90 min. The beads were washed three times with lysis buffer and eluted with 10% SDS and 20% Tris-HCl pH 6.8.

### Pulse and Pulse-Chase Analysis

For total protein synthesis, cells were starved in methionine-free MEM (Sigma) for 30 min and subsequently pulse labeled for 1 h with ^35^S-methionine (0.1 mCi per well in a 6-well plate). Lysates were loaded in a 4%–20% gradient gel, with the amount of lysate normalized for number of cells in each condition. CFTR or HSF1 processing efficiency was measured by pulse-chase. Analysis of CFTR stability by pulse-chase was performed as previously described [56]. For HSF1 pulse-chase, cells were starved in methionine-free MEM (Sigma) for 30 min, pulse labeled for 4 h with ^35^S-methionine (0.1 mCi per well in a 6-well plate), and chased for a total of 24 h. Cells were lysed and HSF1 IP performed as described above. The recovered radiolabeled proteins were then visualized by autoradiography.

### Iodide Efflux Assay

CFBE cells were seeded in 60 mm dishes at a density of 4×10^5^ one day prior to transfection. Iodide efflux assay was performed as previously described [119].

### CFBE-YFP Quenching Assay

CFBE41o- cells stably expressing the halide sensitive YFP-H148Q/I152L [120] (CFBE-YFP), were dosed with compounds 24 h before the YFP-assay, which was performed as previously described [40].

### Transepithelial Short Circuit Current (I_sc_) Measurements

Primary human bronchial epithelial (hBE) cells were dosed every 24 h for a total of 96 h with the indicated concentration of DMSO, VX809, or triptolide. Cells were mounted in modified Ussing chambers, and the cultures were continuously short-circuited with an automatic voltage clamp. Transepithelial resistance, *R*_T_, was measured periodically from the current required to apply a 2.5 mV bipolar voltage pulse. *R*_T_ was calculated from Ohm's law. The basolateral bathing Ringer solution was composed of (137 mM NaCl, 4 mM KCl, 1.8 mM CaCl_2_, 1 mM MgCl_2_, 10 mM HEPES, and 10 mM glucose). NaCl concentration of the apical bathing solution was reduced by replacing NaCl with equimolar Na-gluconate. The chambers were maintained at 37°C and gassed continuously with a mixture of 95% O_2_, 5% CO_2_. Sodium currents were blocked by addition of the sodium channel blocker amiloride (10 µM) to the apical solution. Subsequently, the cAMP agonist, forskolin (10 µM; both chambers), the CFTR potentiator genistein (50 µM; apically), and the CFTR channel blocker CFTRInh-172 (10 µM; apically) were added sequentially to determine cAMP-stimulated CFTR currents.

### Determining CFTR Channel Activity in Human Rectal Organoids

Organoids from a 7-day-old culture (20–80 organoids) were seeded in a 96-well plate (Nunc) in 5 µl Matrigel and 100 µl of medium [77]. One day after seeding, organoids were incubated with 100 µl of medium containing 10 µM calcein green (Invitrogen) for 60 min. Then 5 µM forskolin was added, and organoids were directly analyzed by confocal live-cell microscopy (LSM710, Zeiss, ×5 objective). Three wells were analyzed per condition, and up to 60 wells per experiment. Organoids were pre-incubated for 24 h with 3 µM VX809, 25 nM triptolide, or a combination of both. For CFTR potentiation, 3 µM VX770 was added with forskolin. Organoid surface area was automatically quantified using Volocity imaging software (Improvision). The total organoid surface (XY plane) increase relative to that at T = 0 of stimulus was calculated and averaged from two individual wells per condition. Results are shown as mean ± SD, and p value determined by two-tailed *t*-test using DMSO as a control reference.

### HSF1 Cross-Linking

HSF1 cross-linking to monitor HSF1 trimerization status was performed at room temperature with 1 mM final concentration of disuccinimidyl suberate (DSS) for 30 min with gentle mixing, and quenched by addition of 50 mM Tris-HCl pH 7.5 for 15 min.

### Luciferase Activity Assay

Prior to the luciferase (Luc) assay, cells were lysed and 15 µg of total protein loaded on 8% SDS-PAGE gel to perform immunoblots for Luciferase and actin control to assess Luc expression level. Immunoblots were quantified to ensure that the same amount of Luc was analyzed in the activity assay for each sample. 20 µg of Luc was incubated with Steady-Glo luciferase assay reagent (Promega) for 5 min, and luminescence was read at 562 nm to measure Luc activity. All results are presented as specific FLuc activity, which represents FLuc activity normalized to the amount of FLuc expressed in each condition.

### AAT Secretion

Three hours before measurement of AAT secretion kinetics, cells were washed with PBS and incubated with 350 µl (12-well plate) of FBS-free culture medium. After the 3 h incubation, cells were harvested, and the corresponding media centrifuged at 1500 rpm for 30 min at 4°C to separate cells and medium. After lysis, AAT immature and mature forms in the lysate or secreted into the culture media were analyzed by SDS-PAGE or Native gel for analysis of AAT polymer formation. For native gel electrophoresis, 25 µg of protein in the lysate or 30 µl of cell media was separated on a 3%–20% native gel according to the manufacturer's instructions (Expedeon Inc). Loading of the media was normalized to protein concentration in the lysate for each sample. Native gels were transferred and probed for AAT using the anti-AAT antibody (Immunology Consultants Laboratory).

### AD Mice and Mouse Brain Homogenization

AD mice, referred to as the AβPP Tg mice model, express the hAPP751 cDNA containing the London (V717I) and Swedish (K670M/N671L) mutations under the regulatory control of the murine (m)Thy-1 gene (mThy1-hAPP751). Mice were generated as previously described [121]. For this study, the APP line 41 mice (C57/BI6) were utilized, as they produce high levels of Aβ_42_ and develop synaptic damage and memory deficits. Young (approximately 4 mo old), middle aged (approximately 9 mo old), and old (approximately 16 mo old), WT and AD mice pairs were humanely killed, and tissue was frozen for analysis. Posterior half of mouse hemibrains were homogenized in 500 µl of PDGF buffer (1 mM HEPES, 5 mM Benzamidine, 2 mM 2-Mercaptoethanol, 3 mM EDTA, 0.5 mM Magnesium Sulfate, 0.05% Sodium Azide, 2 mg/ml Protease Inhibitor cocktail [Roche], and 1 tablet of PhosSTOP phosphatase Inhibitor cocktail [Roche] per 10 ml of buffer, pH 8.8), using a tissue homogenizer. Samples were spun at 5,000 g for 5 min at 4°C, and the supernatant centrifuged at 100,000 rpm for 1 h at 4°C to separate the cytosolic and particulated fractions. Pellets were resuspended in 150 µl of PDGF buffer and homogenized by sonication (10% for 10 s). Protein concentration was determined by Bradford, and 20 µg protein from cytosolic fractions were loaded in SDS-PAGE for immunoblotting. To detect Aβ monomer and multimers, 40 µg particulate fractions of brain homogenates were loaded in a 4%–12% bis-tris gel and immunoblots were incubated with 6E10 Aβ specific antibody (Covance).

### CFTR Trypsin Proteolysis

CFBE41o- cells expressing WT- or F508del-CFTR at the indicated treatment were lysed for 30 min at 4°C with lysis buffer (50 mM Tris-HCl pH 7.4, 150 mM NaCl, 1% Triton X-100, 2 mg/ml Protease Inhibitor cocktail [Roche]), and harvested at 20,000 g for 20 min at 4°C. Total protein concentration of pre-cleared lysates was determined by Bradford. Proteolysis was performed by incubating 80 µg of total protein with increasing concentration of Trypsin in PBS (0.01–0.25 mg/ml) at 4°C for 15 min. Proteolysis was stopped by adding 1 mM of PMSF and 6x SDS-PAGE sample buffer. Samples were equally divided and loaded onto two 12% SDS-PAGE for separation of the proteolytic fragments and probed with CFTR antibodies for NBD1 (18D1: epitope 536-545) and NBD2 (M3A7).

### NPC1 Endo-H Digestion

Hela cells expressing WT- or I1061T-NPC1 were transfected for 72 h, lysed in RIPA buffer (10 mM Tris-HCl pH 8.0, 140 mM NaCl, 1 mM EDTA, 1% NP-40, 0.1% SDS, 0.1% Na-deoxycholate, 2 mg/ml Protease Inhibitor cocktail [Roche]), and harvested at 20,000 g for 15 min at 4°C. NPC1 was immunoprecipitated using 400 µg of total protein and 2 µg of NPC1 antibody, for 18 h at 4°C. Complexes were recovered with 40 µl of γ-bind beads incubated at 4°C for 2 h. The beads were washed two times with lysis buffer, and one time with PBS, and eluted with 36 µl of denaturing buffer (NEB) for 10 min at 90°C. Elutions were divided in two tubes, one without and other with 1 µl of endo-H enzyme, and incubate for 1 h at 37°C. Samples were run on 4%–20% gradient gel and immunoblotted for NPC1.

### Data Analysis

The data represents densitometric analysis of immunoblots using an Alpha Innotech Fluorochem SP. The error bars represent the SEM (*n*≥3) or the SD of the mean. In all panels asterisks indicate a *p*-value <0.05 as determined by a two-tailed *t*-test using the control as the reference.

## Supporting Information

Figure S1 **Silencing of F508del-CFTR reduces cellular stress.** (A) Immunoblot and quantification (B) of CFTR, HSF1, and HSF1-P following increased concentration of CFTR siRNA. CFTR immunoblots show the differential migration pattern of CFTR ER localized band-B (lower band), and the post-ER glycoform band-C (higher band). Results are shown as percentage of control (0 nM siCFTR), which is set to 100%. Results are shown as mean ± SD, *n*≥2, and * indicates *p*<0.05 relative to control. The underlying data used to make (B) in this figure can be found in the supplementary file Data S1.
(TIF)

Figure S2 **Silencing of p23 does not affect HSF1 mRNA levels and protein stability.** (A) qRT-PCR of HSF1 levels in F508del-CFTR expressing cells treated with the indicated siRNA. Results represent a ratio of HSF1 to the housekeeping gene GUS and normalized to control siRNA, which is set to 100% (mean ± SD, *n*≥3, * indicates *p*<0.05 relative to control). (B) Pulse chase of HSF1 in response to sip23 treatment. (C) The data is expressed as a percent of HSF1 at time 0 (T = 0). (D) Quantification of the amount of labeled HSF1 during the pulse phase of the pulse-chase described in (B). The data is normalized to control siRNA, which is set to 100% (mean ± SD, *n*≥2, * indicates *p*<0.05 relative to control). (E) Immunoblot of the indicated proteins following p23 silencing in WT-CFTR expressing CFBE cells. Histograms represent quantification of the indicated proteins upon sip23, relative to levels seen with control siRNA, which is set to a 100% (mean ± SEM, *n*≥3, * indicates *p*<0.05 relative to control). The underlying data used to make (A) and (C–E) in this figure can be found in the supplementary file Data S1.
(TIF)

Figure S3 **Silencing of p23 increases F508del-CFTR stability.** Pulse chase analysis of F508del-CFTR (A) and WT-CFTR (C) in response to p23 silencing. CFTR quantification is shown as percent of time 0 (T = 0). Curves were plotted by comparing one-phase exponential decay and linear decay models, and the best fit was chosen. For F508del-CFTR expressing cells the control siRNA data fit one-phase exponential decay (R^2^ = 0.99), whereas the sip23 data was best fit to the linear decay model (R^2^ = 0.97). For WT-CFTR expressing cells control and p23 siRNA data were best fit in one-phase exponential decay. Histograms show the amount of labeled F508del-CFTR (B) or WT-CFTR (D) after the pulse period for the respective pulse-chase experiments shown in (A) and (C) (mean ± SD, *n*≥2, * indicates *p*<0.05 relative to control). (E) Immunoblots of CFTR and p23 levels in response to sip23 treatment in combination with DMSO or the proteasomal inhibitor, MG132 (10 µM for 5 h) in F508del-CFTR expressing cells. Histogram shows the quantitative analysis of F508del-CFTR glycoforms (band-B and band-C) under the indicated conditions. Results were normalized to a percent of the maximum signal for the CFTR band B. Results are shown as a mean ± SD, *n*≥3, and *p*-values determined by two-tailed *t*-test using the indicated condition as reference point; * or # indicate *p*<0.05. (F) Immunoblots of total and ubiquitinated CFTR (poly-Ub) in lysates (input) and CFTR immunoprecipitates (IP:CFTR) in response to sip23 treatment of F508del-CFTR expressing cells. CFTR−/− cells lacking CFTR were used as negative control for CFTR IP. Quantification of CFTR ubiquitination in response to sip23 treatment is shown, as a ratio of ubiquitin to total CFTR and the control siRNA set to 1 (*n* = 3). The underlying data used to make (A–F) in this figure can be found in the supplementary file Data S1.
(TIF)

Figure S4 **HSF1 silencing improves the phenotype of diseases of protein folding.** (A) Quantification of CFTR following HSF1 silencing in F508del-expressing cells. Results are shown as a percentage of maximal band-B for CFTR glycoforms. The C/B ratio is shown as a fold change to that seen in the control condition (set to 1) (mean ± SEM, *n*≥3, * indicates *p*<0.05). (B) Quantification of the indicated proteins in WT-AAT and in the mutant Z-AAT) expressing IB3 cells. The data is shown as a fold change relative to WT-AAT, which is set to 1 (mean ± SD, *n*≥3, * indicates *p*<0.05). Quantitative analysis of immature (I), mature (M) and secreted (S) glycoforms in SDS-PAGE (C) or intracellular and secreted polymers in Native-PAGE (E) of Z-AAT in response to siHSF1 treatment in IB3 cells. Results are shown as fold change relative to control siRNA (mean ± SD, *n*≥3, * indicates *p*<0.05). (D) AAT immunoblots of Native gels, showing intracellular/cytoplasmic (left blot) or secreted (right blot) Z-AAT treated with control or HSF1 siRNA, showing AAT monomer seen on WT-AAT (left arrow) or polymeric forms of Z-AAT (*n* = 3). (F) Quantification of HSF1 and HSF1-P in primary fibroblasts derived from WT- or I1061T-NPC1 patients. Results are shown as fold change relative to WT cells, and represent mean ± SEM, *n*≥3, * indicates *p*<0.05. The underlying data used to make (A–C), (E) and (F) in this figure can be found in the supplementary file Data S1.
(TIF)

Figure S5 **HSF1 silencing is beneficial in a** ***C. elegans*** **model of AD.** (A) Aβ_42_-CFP cDNA construct, which was expressed in transgenic *C. elegans* under the control of the *unc-54* muscle specific promoter. (B) Representative microscopic image showing Aβ_42_ amorphous foci. The white arrow in the right panel indicates Aβ_42_ expression along muscle filaments. Scale bar: I, 50 µm, II, 10 µm. qRT-PCR of the levels of I-Hsp70 (C12C8.1, F44E5.4) after HS in (C) or Aβ_42_ worms in (D) treated with RNAi against YFP (to silence Aβ_42_-CFP) and HSF-1. Results are shown as fold change relative to pre-HS in (C) or as a percentage of control, which is set to 100% in (D) (mean ± SD). Synchronized Aβ_42_ worms were treated daily starting at L4 with control RNAi (L4440) or RNAi against YFP (to silence Aβ_42_-CFP), I-Hsp70 (C12.C8.1 and F44E5.4) (E) or HSF-1 and DAF-2 (F). DAF-2 was used as positive control for increased longevity. Worm mobility was assessed daily for the indicated number of days. Each condition represents data for 100 animals. (G) Quantitative analysis of Aβ_42_-CFP worm paralysis after 7 days in response to HSF-1 overexpression (mean ± SEM). The underlying data used to make (C–G) in this figure can be found in the supplementary file Data S1.
(TIF)

Figure S6 **Silencing of HSF1 and p23 also affect the UPR activation present on F508del-CFTR expressing cells.** (A) qRT-PCR of I-Hsp70 (HspA1A), I-Hsp40 (DNAJB1), I-Hsp90 (Hsp90α), and the stress-responsive small heat shock protein HspB1 (Hsp27), as well as CFTR in F508del-expressing cells after the indicated siRNA treatment. Results represent a ratio of the level of the indicated mRNA to the housekeeping gene GUS and are shown as percentage of control siRNA (* represents *p*<0.05 relative to control siRNA). (B) qRT-PCR of the indicated genes for the unfolded protein response (UPR), ubiquitin proteasomal system (UPS), autophagy, and oxidative stress (NRF2 pathway) PN pathways upon silencing of HSF1 and p23 in F508del-CFTR expressing CFBE cells. Results are shown as fold change relative to control (set to 1, black dotted line) (mean ± SEM, *n*≥3). (C) qRT-PCR comparison of UPR and NRF2 genes in WT- F508del- and CFTR−/− expressing cells. Results are shown as fold change relative to WT (set to 1). For all qRT-PCR experiments, results were normalized to housekeeping gene GUS or TBP (TATA box binding protein), and are shown as mean ± SEM, *n*≥3; * indicates *p*<0.05. (D) Quantification of FLuc activity in WT- or F508del-CFTR expressing cells treated with DMSO, the proteasomal inhibitor MG132 (10 µM for 5 h), the autophagy inhibitor 3-Methyladenide (3-MA, 10 mM for 12 h) or HS at 42°C for 1 h. The data represents normalized FLuc activity (luminescence/relative FLuc expression) for each condition. Results are shown as a mean ± SEM, *n*≥3; * and # indicate *p*<0.05 relative to WT- and F508del-CFTR, respectively. The underlying data used to make (A–D) in this figure can be found in the supplementary file Data S1.
(TIF)

Figure S7 **Alleviation of the MSR by siHSF1 or triptolide can improve the effect of other CF correctors.** (A) qRT-PCR of I-Hsp70 (HspA1A) and I-Hsp90 (Hsp90α) levels after HS of F508del-CFTR expressing cells treated with DMSO or triptolide (12 nM) for 24 h. The data is normalized to the housekeeping gene GUS and is shown as fold change relative to control condition (no HS/DMSO) (mean ± SD, *n*≥2; * indicates *p*<0.05 relative to HS+DMSO). (B) Quantification of CFTR glycoforms (band-B and band-C, left axis) and associated YFP quenching referent to F508del channel activity (right axis) for the indicated treatments of F508del-CFTR expressing CFBE or CFBE-YFP cells. Results are shown as fold change relative to DMSO (set to 1) (mean ± SD, *n*≥3; * indicates *p*<0.05 relative to DMSO). (C) Histogram showing the quenching rates (sec-1) of F508del-expressing CFBE-YFP cells from Fig. 6E. Data represent the mean ± SD, *n*≥3. Quantitative analysis of YFP-quenching in F508del-CFTR expressing CFBE-YFP cells treated with control siRNA or siHSF1, in combination with DMSO or the indicated CF correctors (D) or with the indicated chaperone siRNAs (E). Results are shown as fold change in the percentage of YFP quenching at 30 s relative to control siRNA/DMSO (D) or control siRNA (E) set to 1 (Hdac7 siRNA was used as positive control). Results represent mean ± SD, *n*≥3; * and # indicate *p*<0.05 relative to control siRNA/DMSO and siHSF1/DMSO respectively in (D), or relative to siRNA control in (E). The underlying data used to make (A–E) in this figure can be found in the supplementary file Data S1.
(TIF)

Data S1 **Data used to generate all plots referent to** **Figures 1B, E–G, I–L** **; 2A–E; 3A–F; 4B, F, G; 5A, C, D, F; 6A–E; 7A–C; S1B; S2A, C–E; S3A–F; S4A–C, E, F; S5C–G; S6A–D; S7A–E.**
(XLS)

## Abbreviations

3-MA: 3-methyladenine
AATD: alpha-1-antitrypsin deficiency
AAT: alpha-1-antitrypsin
AD: Alzheimer's disease
APP: Alzheimer precursor protein
BFA: brefeldin A
CF: cystic fibrosis
CFBE: cystic fibrosis bronchial epithelial cells
CFTR: cystic fibrosis transmembrane conductance regulator
CHX: cycloheximide
COPD: chronic obstructive pulmonary disease
ER: endoplasmic reticulum
ERAD: endoplasmic reticulum associated degradation
FLuc: firefly luciferase
GUS: glucoronidase
hBE: human bronchial epithelia
HD: Huntington's disease
HS: heat shock
HSF1: heat shock factor 1
HSR: heat shock response
I-Hsp70: stress inducible Hsp70
I-Hsp90: stress inducible Hsp90
I-Hsp40: stress inducible Hsp40
I_SC_: short circuit current
LE/L: late endosome/lysosome
MSR: maladaptive stress response
NBD: nucleotide binding domain
NPC1: Niemann-Pick type C1 disease
NRF2: nuclear factor erythroid 2-related factor 2
PN: proteostasis network
Q: quinary
siHSF1: small interfering heat shock factor 1
siRNA: small interfering RNA
T1/2: half-life
T2D: type II diabetes
UPR: unfolded protein response
UPS: ubiquitin proteasomal system
YFP: yellow fluorescent protein
Z-AAT: Z-variant of alpha-1-antitrypsin
