# Supplementary figures and images for: Modulation of the Maladaptive Stress Response to Manage Diseases of Protein Folding
Source: PLoS Biol. 2014 Nov 18;12(11):e1001998. doi: 10.1371/journal.pbio.1001998 (PMC4236052; doi:10.1371/journal.pbio.1001998)

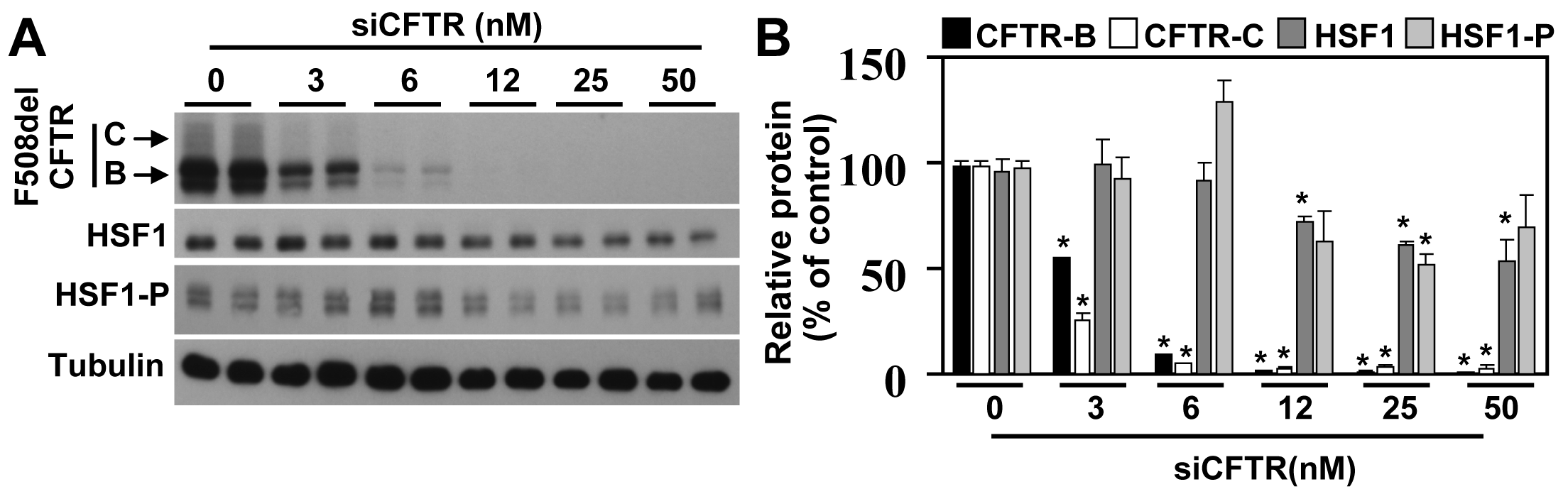

Supplement: Figure S1 — Silencing of F508del-CFTR reduces cellular stress. (A) Immunoblot and quantification (B) of CFTR, HSF1, and HSF1-P following increased concentration of CFTR siRNA. CFTR immunoblots show the differential migration pattern of CFTR ER localized band-B (lower band), and the post-ER glycoform band-C (higher band). Results are shown as percentage of control (0 nM siCFTR), which is set to 100%. Results are shown as mean ± SD, n≥2, and * indicates p<0.05 relative to control. The underlying data used to make (B) in this figure can be found in the supplementary file Data S1. (TIF) [file pbio.1001998.s001.tif]

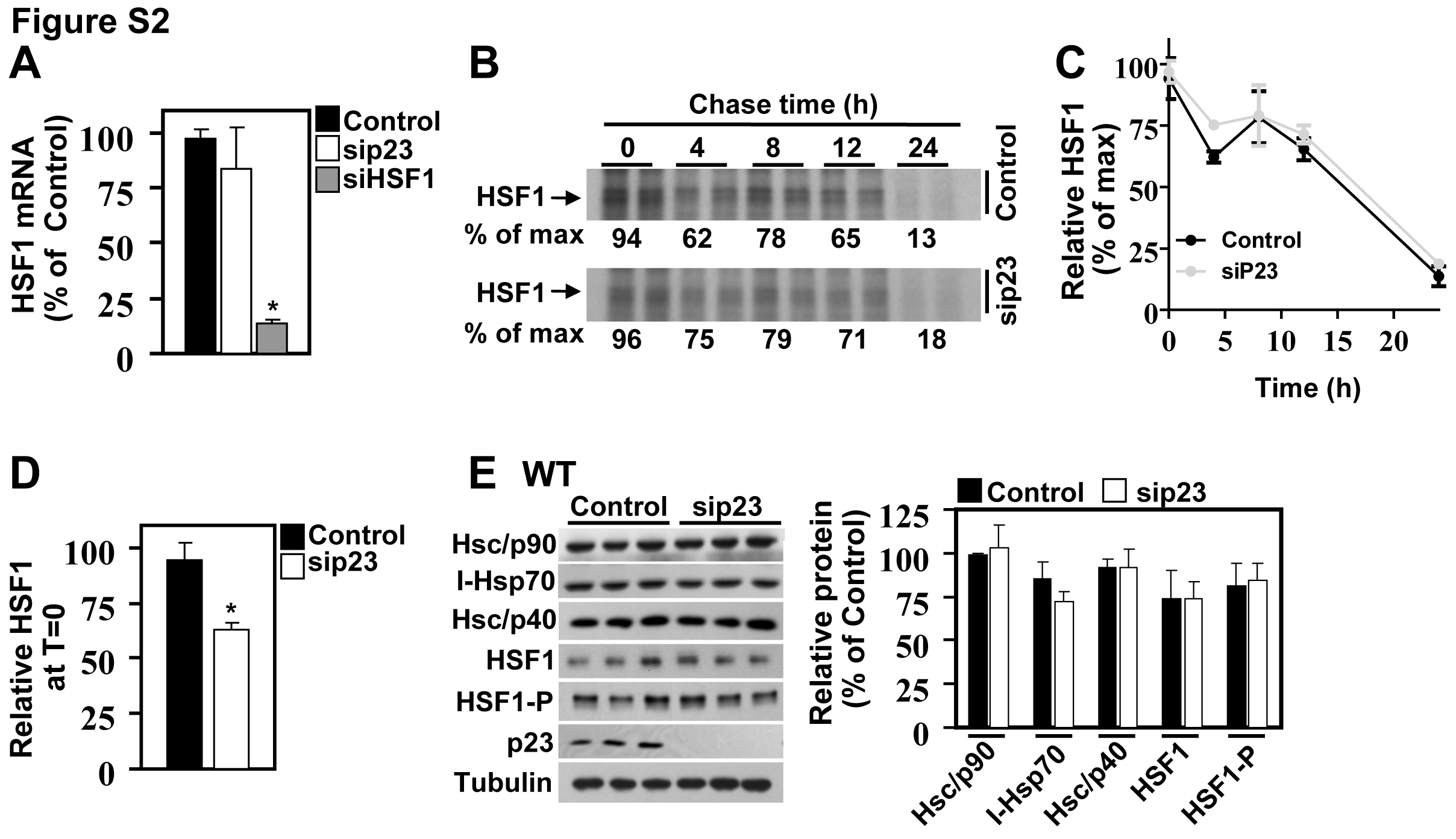

Supplement: Figure S2 — Silencing of p23 does not affect HSF1 mRNA levels and protein stability. (A) qRT-PCR of HSF1 levels in F508del-CFTR expressing cells treated with the indicated siRNA. Results represent a ratio of HSF1 to the housekeeping gene GUS and normalized to control siRNA, which is set to 100% (mean ± SD, n≥3, * indicates p<0.05 relative to control). (B) Pulse chase of HSF1 in response to sip23 treatment. (C) The data is expressed as a percent of HSF1 at time 0 (T = 0). (D) Quantification of the amount of labeled HSF1 during the pulse phase of the pulse-chase described in (B). The data is normalized to control siRNA, which is set to 100% (mean ± SD, n≥2, * indicates p<0.05 relative to control). (E) Immunoblot of the indicated proteins following p23 silencing in WT-CFTR expressing CFBE cells. Histograms represent quantification of the indicated proteins upon sip23, relative to levels seen with control siRNA, which is set to a 100% (mean ± SEM, n≥3, * indicates p<0.05 relative to control). The underlying data used to make (A) and (C–E) in this figure can be found in the supplementary file Data S1. (TIF) [file pbio.1001998.s002.tif]

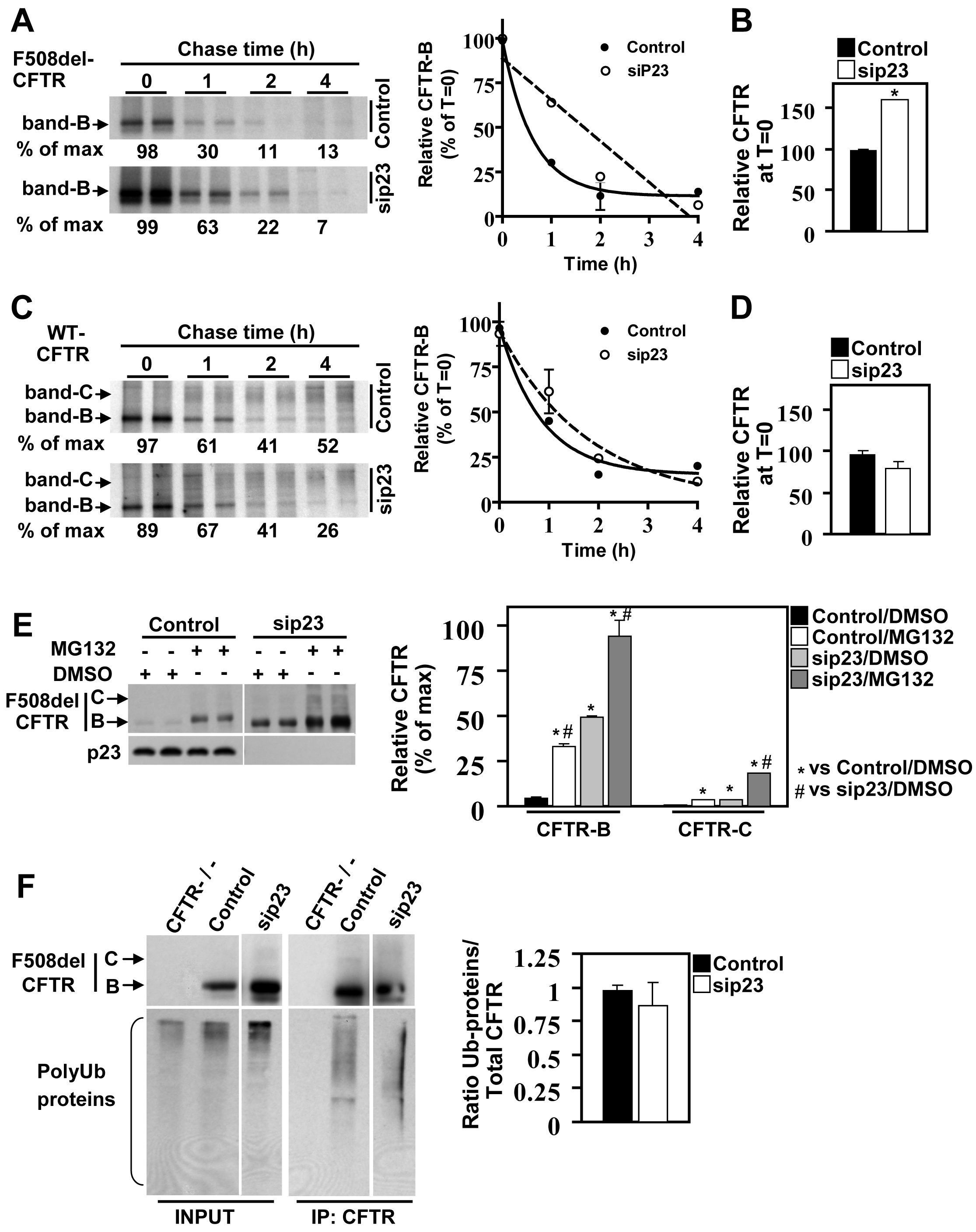

Supplement: Figure S3 — Silencing of p23 increases F508del-CFTR stability. Pulse chase analysis of F508del-CFTR (A) and WT-CFTR (C) in response to p23 silencing. CFTR quantification is shown as percent of time 0 (T = 0). Curves were plotted by comparing one-phase exponential decay and linear decay models, and the best fit was chosen. For F508del-CFTR expressing cells the control siRNA data fit one-phase exponential decay (R2 = 0.99), whereas the sip23 data was best fit to the linear decay model (R2 = 0.97). For WT-CFTR expressing cells control and p23 siRNA data were best fit in one-phase exponential decay. Histograms show the amount of labeled F508del-CFTR (B) or WT-CFTR (D) after the pulse period for the respective pulse-chase experiments shown in (A) and (C) (mean ± SD, n≥2, * indicates p<0.05 relative to control). (E) Immunoblots of CFTR and p23 levels in response to sip23 treatment in combination with DMSO or the proteasomal inhibitor, MG132 (10 µM for 5 h) in F508del-CFTR expressing cells. Histogram shows the quantitative analysis of F508del-CFTR glycoforms (band-B and band-C) under the indicated conditions. Results were normalized to a percent of the maximum signal for the CFTR band B. Results are shown as a mean ± SD, n≥3, and p-values determined by two-tailed t-test using the indicated condition as reference point; * or # indicate p<0.05. (F) Immunoblots of total and ubiquitinated CFTR (poly-Ub) in lysates (input) and CFTR immunoprecipitates (IP:CFTR) in response to sip23 treatment of F508del-CFTR expressing cells. CFTR−/− cells lacking CFTR were used as negative control for CFTR IP. Quantification of CFTR ubiquitination in response to sip23 treatment is shown, as a ratio of ubiquitin to total CFTR and the control siRNA set to 1 (n = 3). The underlying data used to make (A–F) in this figure can be found in the supplementary file Data S1. (TIF) [file pbio.1001998.s003.tif]

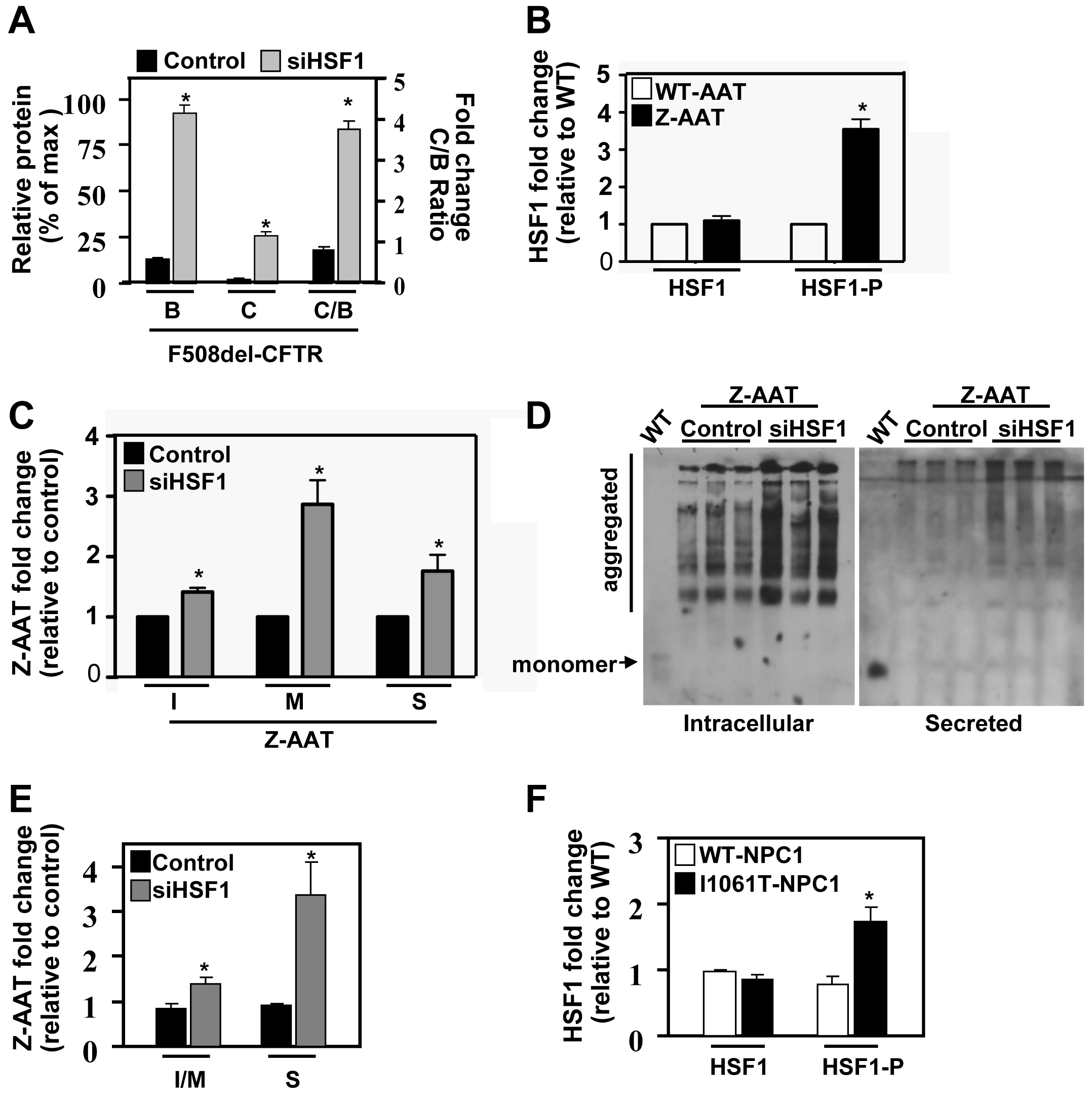

Supplement: Figure S4 — HSF1 silencing improves the phenotype of diseases of protein folding. (A) Quantification of CFTR following HSF1 silencing in F508del-expressing cells. Results are shown as a percentage of maximal band-B for CFTR glycoforms. The C/B ratio is shown as a fold change to that seen in the control condition (set to 1) (mean ± SEM, n≥3, * indicates p<0.05). (B) Quantification of the indicated proteins in WT-AAT and in the mutant Z-AAT) expressing IB3 cells. The data is shown as a fold change relative to WT-AAT, which is set to 1 (mean ± SD, n≥3, * indicates p<0.05). Quantitative analysis of immature (I), mature (M) and secreted (S) glycoforms in SDS-PAGE (C) or intracellular and secreted polymers in Native-PAGE (E) of Z-AAT in response to siHSF1 treatment in IB3 cells. Results are shown as fold change relative to control siRNA (mean ± SD, n≥3, * indicates p<0.05). (D) AAT immunoblots of Native gels, showing intracellular/cytoplasmic (left blot) or secreted (right blot) Z-AAT treated with control or HSF1 siRNA, showing AAT monomer seen on WT-AAT (left arrow) or polymeric forms of Z-AAT (n = 3). (F) Quantification of HSF1 and HSF1-P in primary fibroblasts derived from WT- or I1061T-NPC1 patients. Results are shown as fold change relative to WT cells, and represent mean ± SEM, n≥3, * indicates p<0.05. The underlying data used to make (A–C), (E) and (F) in this figure can be found in the supplementary file Data S1. (TIF) [file pbio.1001998.s004.tif]

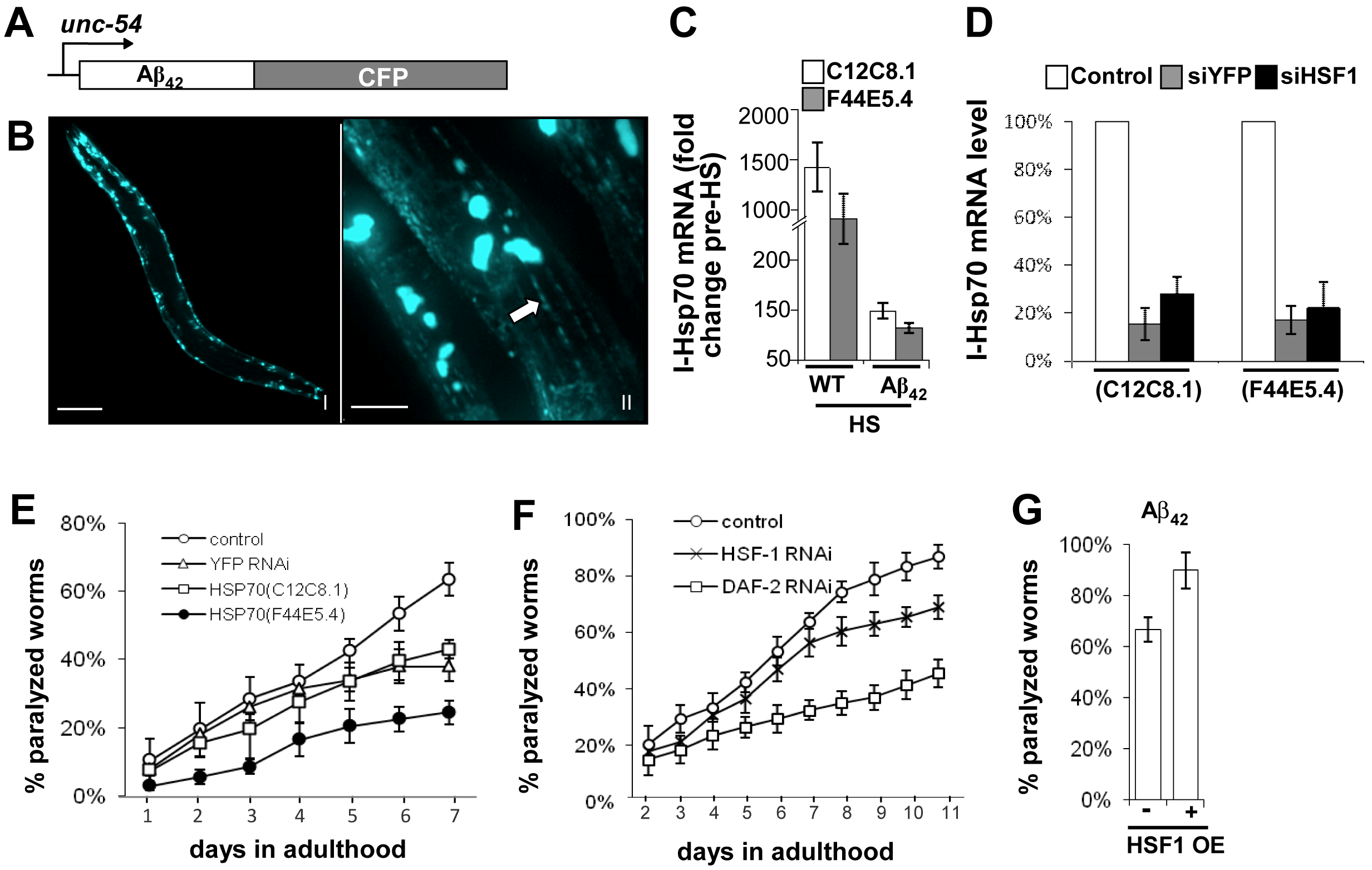

Supplement: Figure S5 — HSF1 silencing is beneficial in a C. elegans model of AD. (A) Aβ42-CFP cDNA construct, which was expressed in transgenic C. elegans under the control of the unc-54 muscle specific promoter. (B) Representative microscopic image showing Aβ42 amorphous foci. The white arrow in the right panel indicates Aβ42 expression along muscle filaments. Scale bar: I, 50 µm, II, 10 µm. qRT-PCR of the levels of I-Hsp70 (C12C8.1, F44E5.4) after HS in (C) or Aβ42 worms in (D) treated with RNAi against YFP (to silence Aβ42-CFP) and HSF-1. Results are shown as fold change relative to pre-HS in (C) or as a percentage of control, which is set to 100% in (D) (mean ± SD). Synchronized Aβ42 worms were treated daily starting at L4 with control RNAi (L4440) or RNAi against YFP (to silence Aβ42-CFP), I-Hsp70 (C12.C8.1 and F44E5.4) (E) or HSF-1 and DAF-2 (F). DAF-2 was used as positive control for increased longevity. Worm mobility was assessed daily for the indicated number of days. Each condition represents data for 100 animals. (G) Quantitative analysis of Aβ42-CFP worm paralysis after 7 days in response to HSF-1 overexpression (mean ± SEM). The underlying data used to make (C–G) in this figure can be found in the supplementary file Data S1. (TIF) [file pbio.1001998.s005.tif]

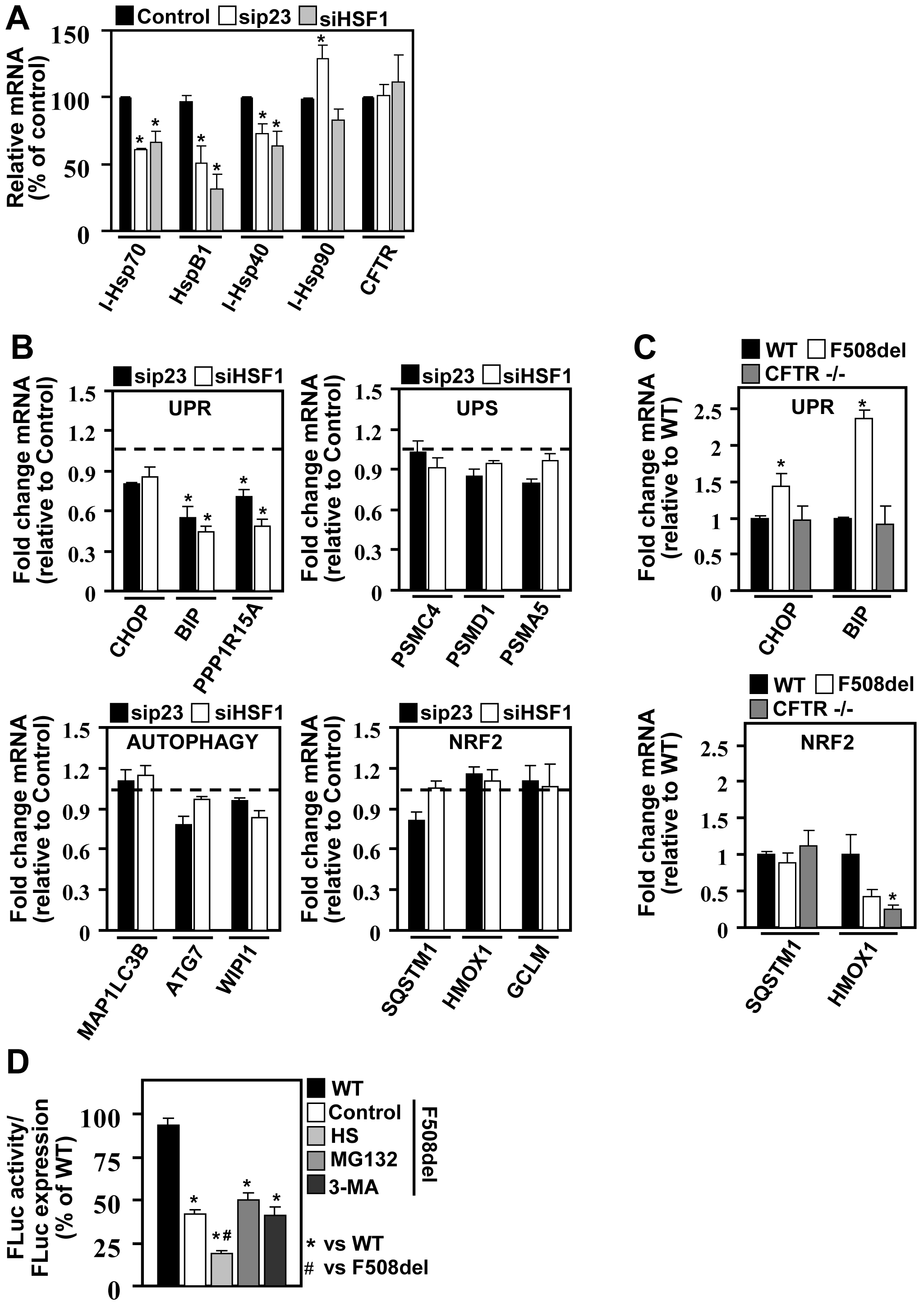

Supplement: Figure S6 — Silencing of HSF1 and p23 also affect the UPR activation present on F508del-CFTR expressing cells. (A) qRT-PCR of I-Hsp70 (HspA1A), I-Hsp40 (DNAJB1), I-Hsp90 (Hsp90α), and the stress-responsive small heat shock protein HspB1 (Hsp27), as well as CFTR in F508del-expressing cells after the indicated siRNA treatment. Results represent a ratio of the level of the indicated mRNA to the housekeeping gene GUS and are shown as percentage of control siRNA (* represents p<0.05 relative to control siRNA). (B) qRT-PCR of the indicated genes for the unfolded protein response (UPR), ubiquitin proteasomal system (UPS), autophagy, and oxidative stress (NRF2 pathway) PN pathways upon silencing of HSF1 and p23 in F508del-CFTR expressing CFBE cells. Results are shown as fold change relative to control (set to 1, black dotted line) (mean ± SEM, n≥3). (C) qRT-PCR comparison of UPR and NRF2 genes in WT- F508del- and CFTR−/− expressing cells. Results are shown as fold change relative to WT (set to 1). For all qRT-PCR experiments, results were normalized to housekeeping gene GUS or TBP (TATA box binding protein), and are shown as mean ± SEM, n≥3; * indicates p<0.05. (D) Quantification of FLuc activity in WT- or F508del-CFTR expressing cells treated with DMSO, the proteasomal inhibitor MG132 (10 µM for 5 h), the autophagy inhibitor 3-Methyladenide (3-MA, 10 mM for 12 h) or HS at 42°C for 1 h. The data represents normalized FLuc activity (luminescence/relative FLuc expression) for each condition. Results are shown as a mean ± SEM, n≥3; * and # indicate p<0.05 relative to WT- and F508del-CFTR, respectively. The underlying data used to make (A–D) in this figure can be found in the supplementary file Data S1. (TIF) [file pbio.1001998.s006.tif]

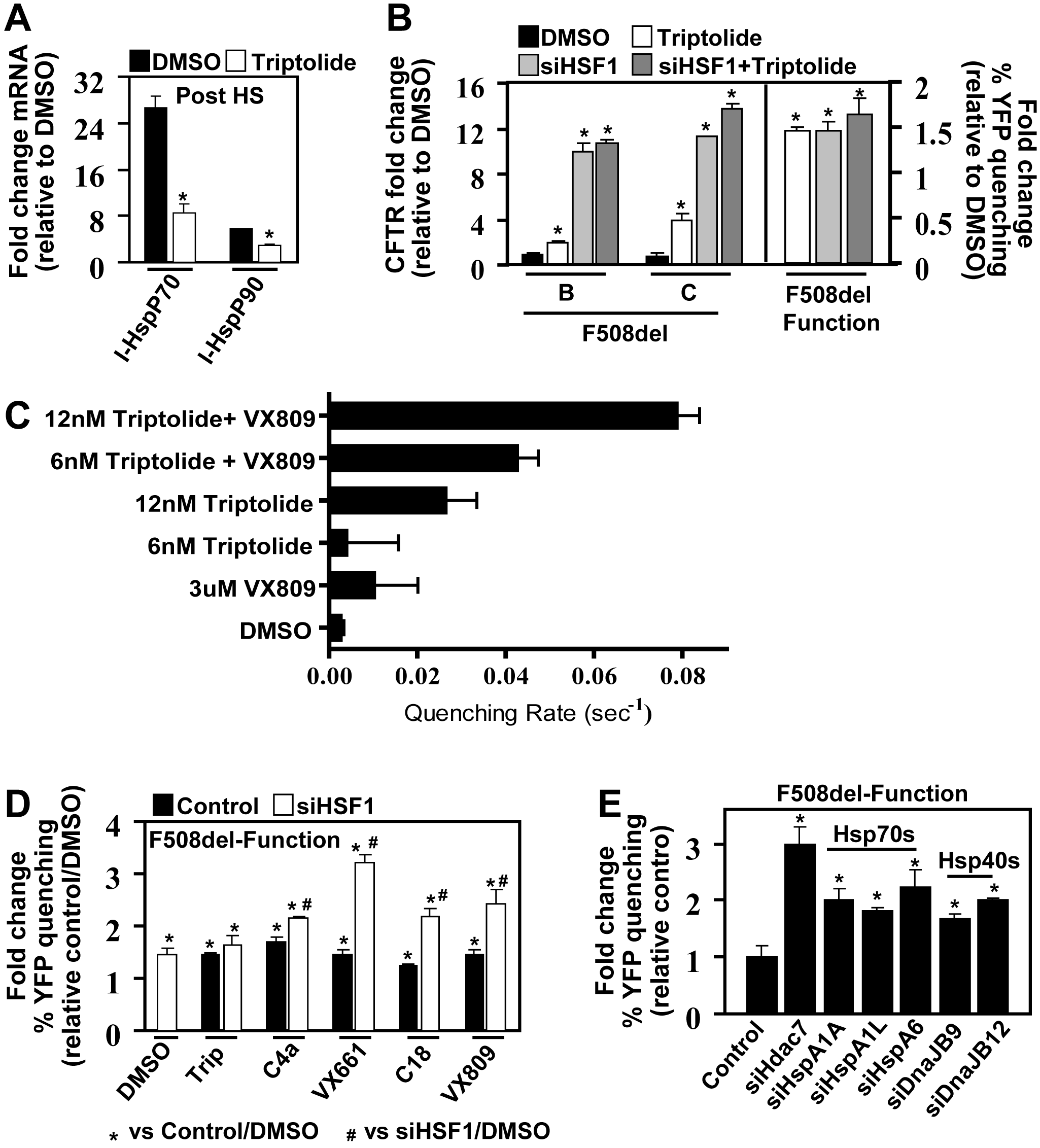

Supplement: Figure S7 — Alleviation of the MSR by siHSF1 or triptolide can improve the effect of other CF correctors. (A) qRT-PCR of I-Hsp70 (HspA1A) and I-Hsp90 (Hsp90α) levels after HS of F508del-CFTR expressing cells treated with DMSO or triptolide (12 nM) for 24 h. The data is normalized to the housekeeping gene GUS and is shown as fold change relative to control condition (no HS/DMSO) (mean ± SD, n≥2; * indicates p<0.05 relative to HS+DMSO). (B) Quantification of CFTR glycoforms (band-B and band-C, left axis) and associated YFP quenching referent to F508del channel activity (right axis) for the indicated treatments of F508del-CFTR expressing CFBE or CFBE-YFP cells. Results are shown as fold change relative to DMSO (set to 1) (mean ± SD, n≥3; * indicates p<0.05 relative to DMSO). (C) Histogram showing the quenching rates (sec-1) of F508del-expressing CFBE-YFP cells from Fig. 6E. Data represent the mean ± SD, n≥3. Quantitative analysis of YFP-quenching in F508del-CFTR expressing CFBE-YFP cells treated with control siRNA or siHSF1, in combination with DMSO or the indicated CF correctors (D) or with the indicated chaperone siRNAs (E). Results are shown as fold change in the percentage of YFP quenching at 30 s relative to control siRNA/DMSO (D) or control siRNA (E) set to 1 (Hdac7 siRNA was used as positive control). Results represent mean ± SD, n≥3; * and # indicate p<0.05 relative to control siRNA/DMSO and siHSF1/DMSO respectively in (D), or relative to siRNA control in (E). The underlying data used to make (A–E) in this figure can be found in the supplementary file Data S1. (TIF) [file pbio.1001998.s007.tif]
